# Characterization of Multitrait Plant Growth‐Promoting Rhizobacteria From *Opuntia ficus‐indica* in Different Moroccan Climates

**DOI:** 10.1002/mbo3.70334

**Published:** 2026-06-23

**Authors:** Ilham Zouitane, Daniela Cristina Campana, Patrizia Cesaro, Nadia Massa, Giorgia Novello, Elisa Gamalero, Valeria Todeschini, Mohamed Ferioun, Khalid Derraz, Saad Ibnsouda Koraichi, Naïma El Ghachtouli, Guido Lingua

**Affiliations:** ^1^ Laboratory of Microbial Biotechnology and Bioactive Molecules, Sciences and Technology Faculty Sidi Mohamed Ben Abdellah University Fez Morocco; ^2^ Department of Science and Technological Innovation University of Eastern Piedmont Alessandria Italy; ^3^ Multidisciplinary Laboratory of Exact and Applied Sciences (LPSEA), Higher School of Technology Fkih Ben Salah Sultan Moulay Slimane University Beni Mellal Morocco; ^4^ Laboratory of Functional Ecology and Environment, Sciences and Technologies Faculty Sidi Mohamed Ben Abdellah University Fez Morocco

**Keywords:** arid environments, cactus pear, drought tolerance, PGPR, phosphate solubilization, sustainable agriculture

## Abstract

Plant growth‐promoting rhizobacteria (PGPR) enhance plant fitness through nutrient mobilization, hormone modulation, and improved tolerance to abiotic stresses. Despite the ecological and agricultural importance of *Opuntia ficus‐indica*, a drought‐adapted cactus widely cultivated in arid and semiarid regions, little is known about its rhizosphere microbiota. This study aimed to select culturable rhizobacteria associated with *O. ficus‐indica*, assessing their plant growth‐promoting (PGP) traits and identifying strains with potential for improving crop performance under challenging environmental conditions. Bacterial strains from the rhizosphere of *O. ficus‐indica* were isolated across three Moroccan regions with distinct rainfall regimes: Tafrant (humid), Fez (semiarid), and Chichaoua (arid). Seventy‐seven strains were isolated and screened for their PGP traits, including phosphate solubilization, indole‐3‐acetic acid, siderophore, ammonia and HCN production, exopolysaccharide synthesis, in vitro nitrogen fixation, and antagonistic activity against *Fusarium solani*. Twenty‐two high‐performing isolates displaying superior PGP traits were selected. Molecular identification revealed taxonomic diversity across three bacterial groups: Gammaproteobacteria (*Pseudomonas*, *Acinetobacter*, *Enterobacter*, and *Stenotrophomonas*), Actinobacteria (*Streptomyces*, *Arthrobacter*, *Kocuria*, and *Citricoccus*), and Firmicutes (*Bacillus*, *Peribacillus*, *Virgibacillus*, *Terribacillus*, and *Priestia*). Isolates from semiarid and arid regions exhibited higher tolerance to drought‐mimicking stress. Several isolates enhanced seed germination and seedling growth parameters in wheat, with the highest germination percentage induced by *Peribacillus frigotolerans* ZFSp2 and *Pseudomonas moraviensis* ZN17. Sorghum germination rate was highest in the presence of ZFSp2. These results highlight the rhizosphere of *O. ficus‐indica* as a valuable reservoir of PGPR with strong potential for applications in sustainable agriculture, particularly in arid and semiarid regions.

## Graphical abstract

The rhizosphere of *Opuntia ficus‐indica* from different Moroccan climates harbors diverse plant growth‐promoting bacteria with multiple beneficial traits. Selected isolates enhanced seed germination and seedling growth, highlighting their potential for sustainable agriculture in arid and semiarid environments.

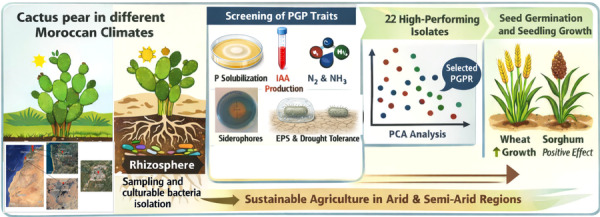

## 1 Introduction

*Opuntia ficus‐indica* (L.) Mill., also named prickly pear or cactus pear, is cultivated in various regions of the world, including Mexico, Countries of the Mediterranean basin, parts of Africa, and the Americas, where it is one of the most economically important species (Kauthale et al. 2017; Martins et al. 2023). This plant is well adapted to arid and semiarid climates and can survive in environments that are inhospitable to other plant species. *O. ficus‐indica* has gained increasing recognition, particularly in Morocco, where new high‐density plantations have been established as part of the Morocco Green Plan (Ramirez and Hernandez 2016). It is a very versatile crop providing food, fodder, and bioactive compounds with significant health benefits (Silva et al. 2021).

The soil directly in contact with plant roots (the rhizosphere) is particularly rich in microorganisms, among which are plant growth‐promoting rhizobacteria (PGPR), which can improve plant fitness (Gamalero and Glick 2015). PGPR can enhance plant growth both directly, by facilitating nutrient acquisition or modulating plant hormone levels, and indirectly, by mitigating abiotic and biotic stresses and acting as biocontrol agents (Ferioun et al. 2024; Glick 1995). These microorganisms also play a crucial role in maintaining soil health and fertility, being involved in ecosystem processes, such as decomposition, mineralization, and nutrient cycling (Ferioun et al. 2025; Nielsen et al. 2015; M. Tahat et al. 2020). Moreover, belowground communities are tightly linked to aboveground ones (Nielsen et al. 2015). For all these reasons, high biodiversity ensures soil health and productivity. Despite its importance, in many regions of the World, soil biodiversity is under threat, due to a variety of causes: excessive land exploitation, climate change, decline in soil organic matter, pollution, soil erosion, land degradation, and loss of aboveground biodiversity (M. Tahat et al. 2020). In arid and semiarid regions, beyond microbial biodiversity, soils are characterized by a scarce vegetative cover, and are therefore highly exposed to erosion by wind and water. These drylands, which cover about 40% of the Earth's surface, are highly vulnerable to various forms of degradation (Arar and Chenchouni 2014; Fitzsimmons et al. 2023; H. Wang et al. 2023). In this context, drought‐adapted plant species represent key ecological resources for maintaining ecosystem functioning and supporting soil microbial communities in drylands. Cactus pear, being resilient to drought, can thrive in these drylands, reducing soil erosion and improving soil structure. Moreover, it is also an ideal plant for intercropping systems: growing cactus pear with other crops not only optimizes land use but also enhances resource efficiency by reducing competition for water and nutrients and improving biodiversity, making it suitable for sustainable farming practices (Jardim et al. 2021; Louhaichi et al. 2017; Nefzaoui 2018).

Native plants from arid and semiarid regions have adapted to survive in harsh conditions thanks to their unique physiological and biochemical traits, representing a valuable resource for improving agricultural resilience (Seleiman et al. 2021; Trovato et al. 2023). In this context, isolating and identifying native PGPR from the rhizosphere of plants such as cactus pear offers promising, environmentally sustainable solutions to enhance soil health and productivity in those regions. Selecting PGPR or other beneficial microorganisms from the rhizosphere of native plants can enhance plant tolerance to salinity and drought, thereby increasing agricultural productivity (Novello et al. 2022).

Targeted inoculation can improve nutrient uptake and plant resilience to abiotic stresses (Ganugi et al. 2019). In fact, PGPR help plants by synthesizing phytohormones, solubilizing nutrients, and producing a beneficial microbial environment (Vuolo et al. 2022). The rational use of microorganisms in agriculture is a promising strategy to improve soil fertility, enhance crop productivity, and promote sustainable farming practices, particularly in regions with low soil fertility or challenges, such as soil degradation, semiarid, or arid conditions (Novello et al. 2022).

In the present work, the authors isolated bacteria from the rhizosphere of *O. ficus‐indica* plants in three regions of Morocco characterized by different rainfall regimes (humid, semiarid, and arid areas). The main aims of this work were to assess the plant‐beneficial physiological traits of the bacterial strains, identify them, and select potential PGPR. These PGPR can then be inoculated onto other crop species cultivated in intercropping systems with *O. ficus‐indica* to promote vegetation in semiarid and arid areas, thereby improving small farmers' income and enhancing soil fertility.

## 2 Materials and Methods

### 2.1 Soil Sampling, Isolation, and Morphological Characterization of Bacteria

#### 2.1.1 Isolation Sites and Soil Sampling

**Figure 1:**
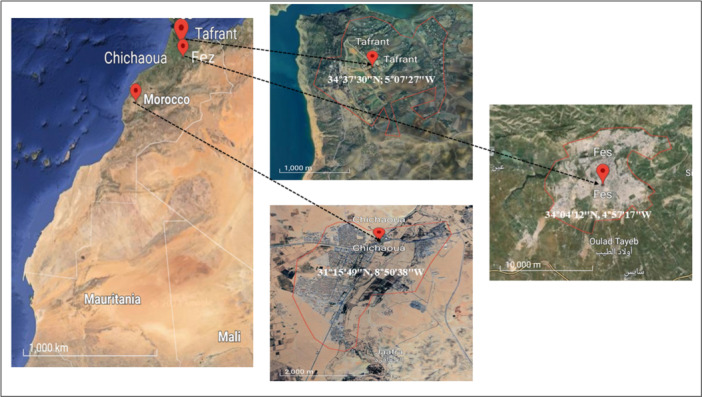
Satellite images of sampling sites of *Opuntia ficus‐indica* rhizospheric soils in Morocco. Tafrant is a humid region (34°37′30″ N, 5°07′27″ W), Fez semiarid region (34°04′12″ N, 4°57′17″ W), and Chichaoua arid region (31°15′49″ N, 8°50′38″ W) (Zouitane et al. 2025).

Bacterial isolation was conducted from three different regions of Morocco: Tafrant humid region (34°37′30″ N, 5°07′27″ W), Fez semiarid region (34°04′12″ N, 4°57'17″ W), and Chichaoua arid region (31°15′49″ N, 8°50′38″ W) (Figure 1). For each region, three independent soil subsamples adhering to the roots of cactus (*O. ficus‐indica*) plants were collected at a depth of 10 cm. The sampling tools were presterilized, and the samples were brought to the laboratory in plastic bags and stored at 4°C for up to 48 h prior to subsequent analyses and bacterial isolation. To obtain a representative overview of the microbial community within a given site, these subsamples were combined into a composite sample at the regional level. Each regional composite sample was treated independently throughout the isolation and screening procedures. Soil samples collected for bacterial isolation were analyzed for their physicochemical characteristics (Zouitane et al. 2025). In the Tafrant region (humid), the soil exhibited a pH of 7.64, electrical conductivity of 226.33 µS/cm, organic matter content of 3.82%, nitrate (NO_3_^−^) concentration of 23.23 µg/g of soil, ammonium (NH_4_^+^) 82.72 µg/g, available phosphorus (P) 123.63 µg/g, available potassium (K) 1670 µg/g, and available iron (Fe) 48.03 µg/g. In the Fez region (semiarid), the soil had a pH of 8.05, electrical conductivity of 290.33 µS/cm, organic matter 5.85%, NO_3_^−^ 80.73 µg/g, NH_4_^+^ 6.89 µg/g, available P 459.60 µg/g, available K 772.00 µg/g, and available Fe 14.48 µg/g. In the Chichaoua region (arid), the soil showed a pH of 7.87, conductivity of 153.33 µS/cm, organic matter content 0.76%, NO_3_^−^ 27.51 µg/g, NH_4_^+^ 6.96 µg/g, available P 58.59 µg/g, available K 2228.00 µg/g, and available Fe 39.94 µg/g.

Soil samples from the rhizosphere of each site were mixed and homogenized in sterile physiological water. Serial decimal dilutions were prepared and used for bacterial isolation by spreading each dilution onto solid Luria–Bertani (LB) agar medium (Lavakush Yadav et al. 2012). To isolate spore‐forming isolates, the dilution series was heated in a water bath at 80°C for 12 min. The inoculated plates were incubated for 48 h at 30°C. Successive streaking of bacteria with different morphological characteristics was performed using the streak plate method. The plates were incubated for another 48 h at 28°C.

#### 2.1.2 Macroscopic and Microscopic Morphological Characterization of Isolates

The macroscopic morphological characterization was determined based on the observation of colony color, opacity, shape, size, elevation, surface, and texture by visual inspection. Gram staining was performed to determine the microscopic morphology of the isolates.

### 2.2 Screening of Rhizospheric Isolates for Plant Growth‐Promoting (PGP) Traits

#### 2.2.1 Inorganic Phosphate Solubilization

The ability of isolates to solubilize inorganic phosphate was tested by inoculating them on solid National Botanical Research Institute's Phosphate (NBRIP) medium containing calcium phosphate (Ca_3_(PO_4_)_2_) as the sole phosphate source (Nautiyal 1999). A 10‐µL aliquot of each isolate was spotted onto the plates, followed by incubation at 28°C for 5 days. The ability of the isolates to solubilize phosphate was indicated by the formation of a clear halo zone around the colonies. The halo zone was quantified by calculating the Phosphate Solubilization Index (PSI) using the following formula: PSI = (Colony diameter + Halo zone diameter)/Colony diameter.

#### 2.2.2 Organic Phosphate Mineralization: Acid and Alkaline Phosphatase Activities

To measure the phosphatase activities of the isolates, bacterial cells, cultured at 30°C for 5 days with shaking at 150 rpm, were washed with a saline solution (0.9% NaCl) and centrifuged. The cells were then resuspended in Tris buffer (pH 7) and sonicated on ice. The supernatant was collected by centrifugation and used to determine phosphatase activities. The phosphatase activities were assayed according to modified protocols of Bradford (1976) and Farhat et al. (2014). The reaction mixture for acid phosphatase contained the enzyme extract in 0.05 M citrate buffer (pH 4.8) with 5.5 mM nitrophenylphosphate. The mixture for alkaline phosphatase contained the enzyme extract in 0.05 M glycine buffer (pH 10.5) with 0.01% MgCl_2_·6H_2_O and 5.5 mM nitrophenylphosphate. The microplates were incubated for 1 h at 37°C. The reactions were stopped by adding NaOH (0.5 N). The amount of *p*‐nitrophenol released was quantified by measuring the absorbance at 405 nm using a microplate reader. A blank (uninoculated LB medium) was run in parallel to account for any spontaneous hydrolysis of 4‐nitrophenyl phosphate during incubation. The results were compared with a standard curve (0.05 µmol/mL of *p*‐nitrophenol).

#### 2.2.3 Indole‐3‐Acetic Acid (IAA) Production

IAA production was determined using the colorimetric method described by Battini et al. (2016). Isolates were inoculated in liquid LB medium supplemented with l‐tryptophan and incubated at 28°C with shaking (200 rpm) for 7 days. The culture was then centrifuged, and the supernatant was mixed with Salkowski's reagent (1.2% FeCl_3_ in 7.9 M sulfuric acid) (Salkowski 1885) and incubated in the dark. IAA production was measured by spectrophotometry at 530 nm. The standard curve was prepared in LB medium containing 0, 10, 25, 50, 75, and 100 μg/mL of IAA (Sigma‐Aldrich) (Chukwuneme et al. 2020).

#### 2.2.4 Exopolysaccharide (EPS) Production

EPS production was assessed using modified rhodospirillum capsulatus V‐sucrose medium (Kaci et al. 2005). Fresh bacterial cultures were inoculated onto plates, which were incubated at 28°C for 7 days. The formation of a gel‐like substance by bacterial colonies on the medium indicated EPS production.

#### 2.2.5 Molecular Nitrogen (N_2_) Fixation

Nitrogen‐fixing bacteria can grow on a nitrogen‐free medium by utilizing atmospheric nitrogen for protein synthesis. Bacterial strains were streaked on Jensen's medium and incubated at 28°C for 8 days (Jensen 1948). Growth on this medium indicated the ability of the bacteria to fix nitrogen.

#### 2.2.6 Ammonia (NH_3_) Production

NH_3_ production was tested in tubes containing sterile peptone water, according to the method of Islam (2009). Cultures were incubated at 28°C for 7 days. Nessler's reagent (10% HgI_2_, 7% KI, and 50% aqueous NaOH solution) was then added to detect ammonia production. A pale‐yellow color indicated low ammonia production, while a dark yellow to brown color signified maximum production.

#### 2.2.7 Siderophore Production

Siderophore production was assessed on King B medium supplemented with Chrome Azurol S, HDTMA, and FeCl_3_ (Schwyn and Neilands 1987). Plates were incubated at 30°C for 72 h. Siderophore‐producing bacteria changed the color of the medium from blue‐green to yellow, forming chelation halos. This color change occurred due to the transfer of ferric ions from the medium to the siderophores. The percentage of siderophore production was estimated based on the formation of halos around bacterial colonies and expressed as a percentage relative to colony diameter using the following formula: % Siderophore = [(halo diameter − colony diameter)/colony diameter] × 100.

#### 2.2.8 Hydrogen Cyanide (HCN) Production

The ability of bacteria to produce HCN was tested according to the method described by Lahlali et al. (2020). Cultures were streaked on yeast extract peptone dextrose medium supplemented with glycine, and a sterile Whatman no. 1 filter paper soaked with picrate solution (2.5% picric acid and 12.5% sodium carbonate) was placed on the Petri dish lid. The dishes were sealed with parafilm and incubated at 28°C for 96 h. HCN production, a volatile compound, was indicated by a color change of the filter paper from yellow to orange.

#### 2.2.9 Antagonistic Effect of Isolates Against the Phytopathogen *Fusarium solani*

The dual culture technique was used to test the antagonistic effect of isolates against the fungus *F. solani* on PDA medium (Ezrari et al. 2021). A plate containing only the fungal mycelial disc served as the control. Plates were incubated at 28°C for 1 week. The presence or absence of an inhibition zone was observed, and the inhibition rate (IR) of mycelial growth was calculated after 1 week of incubation using the following formula: IR (%) = (*C* − *T*)/*C* × 100 (IR = inhibition rate, *C* = fungal colony diameter of the control, and *T* = fungal colony diameter in the presence of the antagonist) (Lahlali et al. 2020).

### 2.3 In Vitro Screening of Drought‐Tolerant Isolates

To assess the ability of bacterial isolates to grow under water stress conditions, LB broth was modified with different concentrations of polyethylene glycol (PEG6000) (0%, 5%, 10%, 20%, and 40%) to achieve varying water potential levels (0, −0.05, −0.15, −0.49, −0.73, and −1.76 MPa, respectively) (Michel and Kaufmann 1973). The medium was then inoculated and incubated at 28°C for 5 days with constant shaking at 150 rpm. Bacterial growth was estimated by measuring optical density at 600 nm using a spectrophotometer (Sandhya et al. 2009).

### 2.4 Molecular Identification of Bacterial Strains

Genomic DNA was extracted using the NucleoSpin microbial DNA purification kit (Macherey‐Nagel, M‐Medical, Cornaredo, Milan, Italy) following the manufacturer's instructions. 16S recombinant DNA (rDNA) PCR amplification was performed using fD1 and rP2 modified primers, as fully described in Gamalero et al. (2020). The length of the 16S ribosomal RNA (rRNA) fragments was 1500 bp. The PCR product was purified using the Nucleo Spin Extract II kit (Macherey‐Nagel) and sequenced by BMR Genomics (Padua, Italy). Electropherograms were analyzed using Finch TV version 4.0 (Applied Biosystems, Milan, Italy) and DNA sequences were blasted against the National Center for Biotechnology Information (NCBI) database using the BLASTn algorithm.

The selected strains were divided into three groups: Gammaproteobacteria, Actinobacteria, and Firmicutes. Phylogenetic trees were constructed using MEGA11 software based on the Maximum Likelihood method and partial 16S rRNA gene sequences. Multiple sequence alignments were performed using the ClustalW algorithm integrated in MEGA, and evolutionary distances were calculated using the Kimura two‐parameter model. The phylogenetic tree was rooted with *Escherichia coli* U 5/41 (DSM 30083). Bootstrap analysis with 500 replicates was performed to evaluate the robustness of the tree branches.

### 2.5 1‐Aminocyclopropane‐1‐Carboxylic Acid (ACC) Deaminase Assay

ZN1 and ZN5 strains, since they were ascribed to the genus *Enterobacter* (thus, potential human pathogens) by molecular identification, were not considered for this test; moreover, ZN8 and ZS7 belonged to the genus *Streptomyces* and so they do not grow similarly to the other strains on the culture, giving unreliable results. The selected 18 bacterial strains were tested for their ACC‐deaminase activity using the Penrose and Glick method (Penrose and Glick 2003). Briefly, bacteria were grown at 28°C for 24 h in Tryptic Soy Broth (TSB medium) and then in salts minimal medium (Dworkin and Foster 1958) with ACC added at a final concentration of 3 mM (Sigma‐Aldrich, Milan, Italy). Then, bacteria were washed with 0.1 M Tris–HCl, pH 7.6, suspended in 0.1 M Tris–HCl, pH 8.5, and in toluene (Sigma‐Aldrich, Milan, Italy). For protein assay, an aliquot of the cells treated with toluene was stored at 4°C. The other aliquot was incubated in the presence or the absence of ACC at 30°C for 15 min. One milliliter of 0.56 M HCl was added, and the suspension was centrifuged at 16,000 g for 10 min at room temperature. Then, 1 mL of the supernatant was added together with 800 µL of 0.56 M HCl and 300 µL of the 2,4‐dinitrophenylhydrazine reagent. After 30 min at 30°C, 2 mL 2 N NaOH was added, and the absorbance of the mixture was measured at 540 nm. *Pseudomonas migulae* 8R6 (Rashid et al. 2012), able to produce ACC deaminase and its mutant, unable to synthesize this enzyme, were used as positive and negative controls, respectively.

### 2.6 Antibiotic Resistance

To test the antibiotic resistance of the bacterial strains, isolates were suspended in 0.1 M MgSO_4_ at an optical density of 0.5 at *λ* 600 corresponding to 10^8^ CFU/mL. Each bacterial suspension (100 µL) was plated onto a Petri dish containing Mueller–Hinton agar medium (Biolife, Milan, Italy). Discs containing the different antibiotics (listed in Table S1) were placed in the center of the Petri dish (one per plate), and the plates were incubated at 28°C for 24 h. Then, the diameter of the inhibition halo around the disc with the antibiotic was measured and this was compared with the standards reported by European Committee on Antimicrobial Susceptibility Testing (EUCAST 2025) to classify the strains as resistant (R), sensitive (S) or intermediate (I). Only 15 strains were tested for antibiotic resistance, since for some of them, there was no EUCAST indication for the type of antibiotic to be employed.

### 2.7 Evaluation of Wheat (*Triticum durum* L.) and Sorghum (*Sorghum bicolor* L.) Seed Germination Following Inoculation With High‐Performing PGPR Isolates (In Vitro)

Germination tests of wheat (*T. durum* L. cv Karim) and leek (*S. bicolor L.*) seeds were conducted to assess the effect of inoculation with high‐performing rhizobacteria (18 isolates) from *O. ficus‐indica*. To ensure the reproducibility and reliability of the results, both germination assays were conducted under controlled laboratory conditions with a constant temperature and maintained in complete darkness to prevent light‐induced physiological variations. The wheat seeds were sterilized using 2% sodium hypochlorite (NaClO) for 1 min, followed by 70% ethanol for 1 min, and then rinsed several times with sterile distilled water to eliminate any traces of ethanol and sodium hypochlorite (Fahsi et al. 2021). The seeds were then immersed in bacterial suspensions for 1 h under agitation. Afterward, 25 seeds were placed in each Petri dish (replicated three times) containing 0.7% sterile agar and incubated at 30°C for 7 days. The *S. bicolor* seeds were sterilized with 1% NaClO for 5 min, then they were rinsed with sterile deionized water for six times. The seeds were maintained in agitation (180 rpm) for 40 min in 0.1 M MgSO_4_ (for controls) or in bacterial inoculum (0.5 OD600) in 0.1 M MgSO_4_ (for treatments). The seeds were then placed in 15‐cm Petri dishes on a paper imbibed with sterilized deionized water (three replicas for each treatment). The plates were incubated in the dark at 24°C. Germinated seeds were counted, and growth parameters, such as shoot and root lengths, fresh and dry weights of shoots and roots, and the number of roots were measured for each Petri dish. The germination percentage (GP%), germination rate index (GRI), seedling length vigor index (SLVI), and seedling weight vigor index (SWVI) were calculated using the following equations (Kerbab et al. 2021):

– GP% = (Number of germinated seeds/Total number of seeds) × 100.
– GRI = G2/2 + G3/3 + G4/4 (G2, G3, and G4 were germination percentages at 2, 3, and 4 days).
– SLVI = Seedling length (cm) × GP%.
– SWVI = Seedling dry weight (mg) × GP%.

### 2.8 Data Analysis

All in vitro assays were conducted following a completely randomized design, where bacterial isolates were randomly assigned to experimental units. The obtained data were shown as mean ± standard deviation (three independent biological replicates (*n* = 3), and appropriate statistical analyses were performed to assess differences among treatments. Each experiment was repeated independently to ensure reproducibility. Tukey test was applied using Minitab 21.2 software (64‐bit), both for the PGP traits of the bacterial isolates and for the germination parameters of wheat and sorghum seeds. Differences were considered statistically significant at *p* ≤ 0.05. For the data on seed germination, normality was assessed using the Shapiro–Wilk test, and homogeneity of variances was evaluated using Levene's test.

Principal component analysis (PCA) was performed using a Pearson correlation matrix, which inherently standardizes the variables by removing scale effects. This approach was chosen because the analyzed PGP traits were measured in different units and ranges, making correlation‐based PCA more appropriate than covariance‐based PCA. PCA was carried out with XLSTAT 2016.02.28451 software to select the high‐performing isolates based on their PGP traits.

## 3 Results

### 3.1 Bacterial Isolates and Morphological Characterization

**Figure 2:**
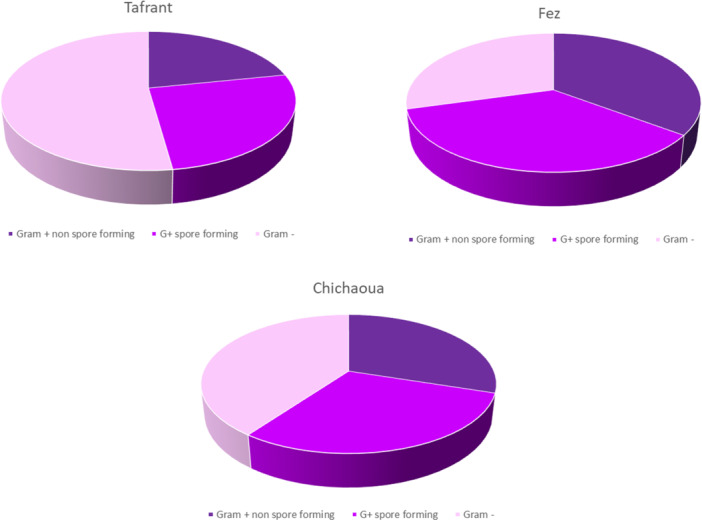
Proportion of Gram‐negative, spore‐forming Gram‐positive, and non‐spore‐forming Gram‐positive bacteria in the rhizosphere of *Opuntia ficus‐indica* grown in the three sites.

A total of 77 isolates were initially selected from the rhizosphere of *O. ficus‐indica*. According to the isolation site, 23 strains were from the Tafrant humid region, 34 isolates from the Fez semiarid region, and 20 isolates were collected from the Chichaoua arid region. On the basis of their morphological characteristics, most of the colonies exhibited smooth surfaces, mucoid consistency, round shapes, regular margins, variable sizes, and diverse pigmentation. Microscopic observation revealed that 28.57% of the isolates were cocci‐shaped, 46.75% were rod‐shaped, 18.18% were coccobacilli‐shaped, and 6.50% were filamentous. Regarding the reactivity to Gram staining, 12 out of 23 (52.2%) of the bacterial strains isolated from the Tafrant region were Gram negative, while 11 (47.8%) were Gram positive. Six Gram‐positive strains were classified as spore‐forming (Table S2). A different situation was observed in the Fez region, where 10 out of 34 (29.4%) of the bacterial strains were Gram negative and 24 (70.6%) were Gram positive. Half of the Gram positive (12 isolates) were spore‐forming (Figure 2 and Table S2). Finally, of the 20 isolates collected from the Chichaoua arid region, eight (40%) were Gram negative and 12 (60%) were Gram positive; six of the Gram‐positive strains were spore‐forming (Figure 2 and Table S2).

### 3.2 PGP Traits of Bacterial Isolates From *O. ficus‐indica* Rhizosphere

All tested isolates exhibited multiple PGP traits, as evidenced by the results of the qualitative and quantitative PGP characteristics (Table S3).

*Phosphate solubilization:* Overall, 20.78% of the isolates (16 out of 77) demonstrated phosphate solubilization activity by forming solubilization halos of varying sizes on NBRIP‐agar medium. To obtain quantitative information on phosphate solubilization, both acid, and alkaline phosphomonoesterase were measured (expressed in µmol of PNP/h/mL at pH 4.8 for acid or pH 10.5 for alkaline phosphatase, at 37°C) and the results are presented in Table S3. The results show that acid phosphatase activity was detected in only 18.18% of the isolates, whereas alkaline phosphatase activity was present in 90.91% of the isolates, with significant variability. Acid phosphatase activity ranged from 0.1538 µmol/h/mL (isolate ZN1) to 0.0001 µmol/h/mL (isolate ZN13), while alkaline phosphatase activity ranged from 0.6989 µmol/h/mL (isolate ZS1) to 0.0006 µmol/h/mL (isolate ZS8). Isolates ZN2, ZN7, ZN14, and ZNSp1 from the Tafrant region, along with isolates ZF2, ZF9, and ZF21 from the Fez region, showed no detectable acid or alkaline phosphatase activities. According to the results presented in Table S3, most isolates from the Tafrant region exhibited higher acid phosphatase activity than alkaline phosphatase activity, while isolates from the Fez and Chichaoua regions displayed the opposite trend.

*IAA synthesis:* According to the data presented in Table S3, 44.16% (34 isolates) of the tested isolates produced IAA, with concentrations ranging from 1.63 µg/mL (isolate ZFSp12) to 25.98 µg/mL (isolate ZS6A). The lowest percentage of IAA‐producing strains (35.29%, 12 isolates) was recorded in the Fez region, with production not exceeding 13.25 µg/mL (strain ZF12). Ten bacterial strains out of 33 (43.48%) from the Tafrant region were able to synthesize IAA, with a maximum concentration of 23.43 µg/mL (strain ZN5). The highest percentage of IAA‐producing strains was observed in the Chichaoua region, where 60.% of the isolates released IAA, with a maximum concentration of 25.98 µg/mL (strain ZS6A).

*Nitrogen fixation and ammonia production:* Overall, 50.65% of the bacterial isolates (39 strains) were able to fix atmospheric nitrogen. The proportion of nitrogen‐fixing bacteria was different in the three sites: 52.17% (12 isolates) from the Tafrant region, 35.29% (12 isolates) from the Fez region, and 75.00% (15 isolates) from the Chichaoua region (Table S3). The isolates were also tested for their ability to produce ammonia: overall, 58.44% (45 isolates) of the bacterial isolates was able to produce ammonia. Of these, 78.26% of isolates from the Tafrant region and 50.00% from the Fez and Chichaoua regions showed positive results (Table S3).

*Siderophore synthesis:* Table S3 shows the ability and proportion of isolates capable of producing siderophores. Overall, 74.03% (57 isolates) of the isolates produced siderophores. The isolates from the Tafrant region exhibited a production rate of 65.22% (15 isolates), followed by those from the Fez region at 76.47% (26 isolates), and finally those from the Chichaoua region at 80.00% (16 isolates). The highest siderophore producer was strain ZS7 isolated from the Chichaoua region, with a percentage of 315.11%.

*Cyanide production:* The bacterial isolated in this study were tested for their ability to produce HCN, and the results are presented in Table S3. Overall, 18.18% (14 strains) of the isolates were able to synthesize HCN. Among them, 26.09% (six strains) of the isolates from the Tafrant region, 20.00% (four strains) from Chichaoua, and 11.76% (four strains) from Fez produced HCN.

*Antagonistic activity against F. solani:* The antagonistic potential of the isolates against the mycelial growth of *F. solani* is shown in Table S3. After 1 week of incubation in the presence of the bacterial strains, the results showed varying IRs of mycelial growth depending on the isolates. Two out of 23 isolates (ZN4 and ZNSp5) from the Tafrant region reduced the in vitro mycelial growth of *F. solani*, with IRs overcoming 60.00%. Fifteen isolates exhibited inhibition ranging from 8.57% to 50.00%, while six isolates did not show antagonistic ability against the phytopathogenic fungus. Among the 34 strains isolated from the Fez region, one (ZFSp6) demonstrated an IR exceeding 60.00%, 18 showed inhibition ranging from 12.12% to 50.28%, and 15 were ineffective. Regarding the Chichaoua isolates, two (ZS7 and ZSSp5) out of 20 induced growth inhibition above 60%, 11 showed inhibition ranging from 14.26% to 59.61%, and seven isolates failed to reduce the mycelial growth of *F. solani*.

*EPS production and Drought tolerance:* According to the results shown in Table S3, 45.45% of the isolates (35 strains) were able to produce EPS. The isolates from the Tafrant region showed the highest proportion of EPS producers (52.17%), followed by isolates from Chichaoua (50.00%), and finally those from Fez (38.24%).

The ability of isolates to withstand water stress induced by PEG6000 was also evaluated, and the results are presented in Tables 1 and S3. Overall, 48 out of 77 bacterial strains, 15 (65%) isolated from Tafran, 22 (64.7%) from Fez and 11 (55%) from Chichaoua were able to grow in the presence of PEG6000. Four bacterial strains (corresponding to 5.19%) were able to grow at a water potential of −0.73 MPa, while 12.99% (10 strains), 29.87% (23 strains), and 14.29% (11 strains) of the selected isolates were able to grow at water potentials of −0.49, −0.15, and −0.05 MPa, respectively. In the semiarid (Fez) and arid (Chichaoua) regions, a higher proportion of bacteria tolerant to water potential below –0.49 MPa (23.53% and 15.00%, respectively) compared with the humid region (8.70%) was observed. Finally, 29 bacterial strains (corresponding to 37.66%), eight from Tafrant, 12 from Fez, and nine from Chichaoua, showed complete intolerance to PEG6000.

**Table 1:** Plant growth‐promoting traits of *Opuntia ficus‐indica* rhizobacteria.

Isolates	PSI	Acid phosphatase (µmol PNP/h/mL)	Alkaline phosphatase (µmol PNP/h/mL)	IAA (µg/mL)	EPS	N_2_	NH_3_	Siderophore (%)	HCN	Antagonism index (IR %)	Drought stress (MPa)
ZN1	3.96 ± 0.35^b^	0.1538 ± 0.0011^a^	0.0806 ± 0.0010^f^	5.69 ± 0.79 ^fg^	+	+	+	ND	+	32.54 ± 0.89^f^	−0.05 ± 0.00^d^
ZN3	4.38 ± 0.41^a^	ND	0.0106 ± 0.0005^rstuvw^	10.09 ± 0.59^d^	−	+	+	23.22 ± 0.59^no^	+	31.11 ± 0.47^f^	− 0.15 ± 0.00^c^
ZN4	3.78 ± 0.21^b^	0.0334 ± 0.0006^e^	0.0108 ± 0.0000^qrstuv^	4.89 ± 0.15^fghi^	−	−	+	26.18 ± 1.17^mn^	+	62.69 ± 0.84^ab^	−0.05 ± 0.00^d^
ZN5	2.79 ± 0.04^d^	0.0041 ± 0.0001^gh^	0.0083 ± 0.0001^stuvwx^	23.43 ± 1.11^a^	−	−	+	13.88 ± 0.28^qr^	−	53.33 ± 5.77^c^	ND
ZN8	2.78 ± 0.17^d^	0.0735 ± 0.0044^c^	0.3160 ± 0.0096^b^	1.28 ± 0.13^opq^	−	+	+	52.77 ± 0.51^i^	−	26.43 ± 0.52^g^	ND
ZN11	ND	ND	0.3390 ± 0.0106^a^	0.95 ± 0.02^opq^	+	−	+	100.00 ± 0.00^d^	−	50.00 ± 0.29^cde^	ND
ZN12	1.18 ± 0.07^gh^	0.0032 ± 0.0002^gh^	0.1161 ± 0.0050^e^	6.03 ± 0.21^ef^	+	+	+	58.28 ± 0.38^h^	−	33.97 ± 1.26^f^	−0.15 ± 0.00^c^
ZN17	3.45 ± 0.17^c^	0.0240 ± 0.0005^e^	0.0092 ± 0.0001^rstuvw^	3.36 ± 0.49^jkl^	+	−	+	8.91 ± 1.01^st^	+	16.03 ± 0.57^ij^	−0.73 ± 0.00^a^
ZNSp3	1.29 ± 0.04^gh^	ND	0.0148 ± 0.0009^opqrs^	0.51 ± 0.04^pq^	+	+	+	100.00 ± 2.85^d^	+	50.00 ± 1.17^cde^	−0.15 ± 0.00^c^
ZF1	1.67 ± 0.13^f^	ND	0.0149 ± 0.0004^opqrs^	0.68 ± 0.03^pq^	+	+	+	17.48 ± 1.48^pq^	−	46.76 ± 0.99^e^	−0.05 ± 0.00^d^
ZF4B	2.50 ± 0.20^de^	0.0849 ± 0.0001^b^	0.2586 ± 0.0059^c^	0.51 ± 0.03^pq^	+	+	+	6.11 ± 0.32^t^	+	33.33 ± 1.22^f^	ND
ZF11	ND	ND	0.0036 ± 0.0000^wxyz^	10.50 ± 0.74^cd^	+	+	+	100.00 ± 1.80^d^	−	33.08 ± 0.50^f^	−0.49 ± 0.00^b^
ZF13	ND	0.0034 ± 0.0001^gh^	0.0066 ± 0.0000^uvwxyz^	ND	−	−	−	249.66 ± 1.11^c^	−	16.66 ± 1.63^ij^	−0.49 ± 0.00^b^
ZF16	ND	ND	0.0324 ± 0.0006^ij^	5.71 ± 0.11^fg^	−	−	−	272.22 ± 6.05^b^	−	12.12 ± 0.73^kl^	−0.49 ± 0.00^b^
ZFSp2	ND	ND	0.0140 ± 0.0002^pqrst^	11.69 ± 0.74^c^	+	+	+	ND	−	ND	−0.49 ± 0.00^b^
ZFSp5	1.12 ± 0.13^h^	ND	0.0687 ± 0.0003^g^	2.87 ± 0.33^klmn^	+	+	+	22.95 ± 0.99^no^	−	53.24 ± 1.27^c^	−0.73 ± 0.00^a^
ZS7	ND	ND	0.0107 ± 0.0004^qrstuvw^	2.29 ± 0.54^lmno^	−	+	−	315.11 ± 0.19^a^	−	60.06 ± 0.41^b^	−0.15 ± 0.00^c^
ZS8	ND	ND	0.0006 ± 0.0000^yz^	18.11 ± 0.50^b^	−	+	−	89.79 ± 2.14^ef^	+	ND	−0.05 ± 0.00^d^
ZSC	ND	0.0117 ± 0.0150^f^	0.0350 ± 0.0002^hi^	9.94 ± 0.50^d^	+	+	+	47.92 ± 0.88^ij^	−	14.26 ± 0.35^jk^	−0.15 ± 0.00^c^
ZSSp2	1.42 ± 0.06^fg^	ND	0.1278 ± 0.0031^d^	ND	+	+	+	31.06 ± 1.11^klm^	−	48.33 ± 0.67^de^	−0.49 ± 0.00^b^
ZSSp3	1.24 ± 0.05^gh^	ND	0.1188 ± 0.0011^e^	3.11 ± 0.50^jkl^	+	+	+	ND	−	59.61 ± 1.18^b^	−0.15 ± 0.00^c^
ZSSp5	ND	ND	0.0227 ± 0.0004 ^klmn^	22.64 ± 0.51^a^	−	+	−	84.94 ± 0.92^fg^	+	62.69 ± 1.36^ab^	−0.05 ± 0.00^d^

*Note:* Mean + Standard errors. Scores with different letters indicate significant differences among bacterial strains based on the Tukey test (*p* < 0.05).

Abbreviations: −, negative test; +, positive test; EPS, exopolysaccharides; HCN, hydrogen cyanide; IAA, indole‐3‐acetic acid; IR, inhibition rate; N_2_, nitrogen; ND, not detected; NH_3_, ammonia; PNP, *p*‐nitrophenyl; PSI, phosphate solubilization index.

### 3.3 Selection of High‐Performing Isolates in Terms of PGP Traits Using PCA

**Figure 3:**
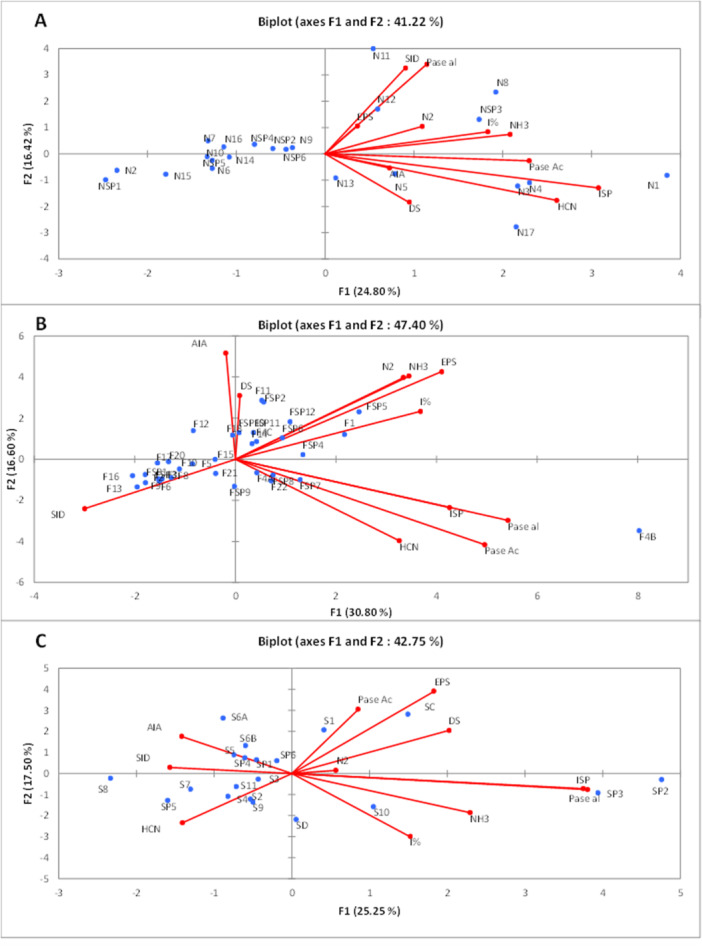
Principal component analysis of PGP traits of rhizospheric isolates from *Opuntia ficus‐indica* in each region (Tafrant region A, Fez region B, and Chichaoua region C). DS, drought stress resistance; EPS, exopolysaccharide; HCN, hydrogen cyanide production; I%, percentage inhibition of *Fusarium solani*; IAA, indole acetic acid production; ISP, phosphate solubilization index; N_2_, nitrogen fixation; NH_3_, ammonia production; Pase ac, acid phosphatase activity; Pase al, alkaline phosphatase activity; SID, siderophore production.

PCA is used to explore multidimensional data sets with quantitative variables. In this study, PCA was employed to compare the quantitative PGP activities exhibited by different isolates from each region, allowing for the selection of the most effective isolates in terms of plant‐beneficial features (Figure 3A–C). In the Tafrant region, the PCA revealed two principal components (eigenvalue ≥ 1) accounting for 41.22% of the total variability. The PCA plot (Figure 3A) discriminates the ZN1 isolate, which exhibited high acid phosphatase activity, better production of hydrogen cyanide, and inorganic phosphate solubilization in the positive section of F1. Isolates ZN8 and ZNSp3 demonstrated strong performance in nitrogen fixation, ammonia production, and antagonistic activity against the phytopathogenic fungus *F. solani*. Additionally, isolate ZN17 stood out as the best in terms of IAA production and drought stress resistance. EPS production, siderophore production, and alkaline phosphatase activity characterized isolates ZN11 and ZN12, with intermediate values, also observed in isolate ZN5 for indole acetic acid production. The other isolates from the region showed minimal values for the PGP traits studied.

Considering the strains isolated from the Fez Region, the PCA plot (Figure 3B), constructed with the first two principal components F1 and F2, accounted for 47.40% of the total variability.

Isolate ZF4B was distinctly positioned in the positive section of F1, showing high values for inorganic phosphate solubilization and both acid and alkaline phosphatase activities. Isolates ZF1 and ZFSp5 demonstrated intermediate production of EPS, NH_3_, nitrogen fixation, and antagonistic activity against *F. solani*. Drought stress resistance was observed in the positive F2 section for isolates ZF11 and ZFSp2. The negative section of F1 and F2 highlighted isolates ZF13 and ZF16 due to their siderophore production.

Finally, regarding the strains isolated from the Chichaoua Region, the two principal components, F1 and F2, formed the PCA plot with a total variability of 30.80% (Figure 3C). In the positive section of F1, isolates ZSSp2 and ZSSp3 were discriminated due to their strong alkaline phosphatase activity and efficient inorganic phosphate solubilization. Isolate ZSC showed intermediate EPS production and drought stress resistance in the positive section of F1 and F2. The negative section of F1 distinguished isolates ZS7, ZS8, and ZSSp5 by their production of siderophores and hydrogen cyanide.

A total of 22 putatively high‐performing isolates were selected based on their position in the PCA space, prioritizing those located at the periphery of the ordination plot, as well as those associated with high values of key PGP traits: nine from the Tafrant region (ZN1, ZN3, ZN4, ZN5, ZN8, ZN11, ZN12, ZN17, and ZNSp3), seven from the Fez region (ZF1, ZF4B, ZF11, ZF13, ZF16, ZFSp2, and ZFSp5), and six from the Chichaoua region (ZS7, ZS8, ZSC, ZSSp2, ZSSp3, and ZSSp5) (Table 1).

### 3.4 Molecular Identification of Bacterial Strains

The results about the molecular identification of the 22 high‐performing bacterial strains (nine from Tafrant, seven from Fez, and six from Chichaoua regions) are reported in Table 2. The bacterial 16S rDNA reference sequences of these isolates are available at the NCBI World Wide Web database GenBank, as SUB 15320870, with the accession numbers reported in Table 2.

**Table 2:** Molecular identification of bacterial strains.

Isolates	Scientific name	Sequence coverage^a^ (%)	Identity^b^ (%)	Sequence ID^c^	NCBI Accession number^d^
ZN1	*Enterobacter hormaechei*	97	99.41	CP058146.1	PV639455
ZN3	*Pseudomonas frederiksbergensis*	100	99.27	CP017886.1	PV639456
ZN4	*Pseudomonas koreensis*	99	99.60	MN685252.1	PV639457
ZN5	*Enterobacter* sp.	97	98.54	KC413033.1	PV639458
ZN8	*Streptomyces endophyticus*	97	99.11	MN826178.1	PV639459
ZN11	*Stenotrophomonas* sp.	99	99.42	PP094573.1	PV639460
ZN12	*Bacillus halotolerans*	100	99.70	CP098738.1	PV639461
ZN17	*Pseudomonas moraviensis*	99	99.69	CP099608.1	PV639462
ZNSp3	*Bacillus pumilus*	100	99.60	KR140174.1	PV639463
ZF1	*Bacillus safensis*	100	99.80	MK184539.1	PV639464
ZF4B	*Bacillus cereus*	100	99.89	MT394427.1	PV639465
ZF11	*Arthrobacter globiformis*	99	99.49	MF620067.1	PV639466
ZF13	*Virgibacillus proomii*	100	99.28	FN397532.1	PV639467
ZF16	*Kocuria carniphila*	100	99.61	ON693794.1	PV639468
ZFSp2	*Peribacillus frigoritolerans*	100	99.60	KY753329.1	PV639469
ZFSp5	*Bacillus subtilis*	100	99.89	MK312473.1	PV639470
ZS7	*Streptomyces venezuelae*	99	100	KY753284.1	PV639471
ZS8	*Citricoccus* sp.	97	99.37	PP778114.1	PV639472
ZSC	*Terribacillus aidingensis*	99	99.21	AB748966.1	PV639473
ZSSp2	*Bacillus halotolerans*	100	99.80	PQ410718.1	PV639474
ZSSp3	*Priestia megaterium*	100	99.35	ON932305.1	PV639475
ZSSp5	*Acinetobacter johnsonii*	99	98.85	OM232065.1	PV639476

^a^ BLAST sequence coverage obtained by comparing each bacterial 16S recombinant DNA (rDNA) sequence to all the reference sequences available at the National Center for Biotechnology Information (NCBI).

^b^ BLAST identity results obtained by comparing each bacterial 16S rDNA sequence to all the reference sequences available at the NCBI.

^c^ Reference sequences available at the NCBI.

^d^ Accession numbers of the deposited sequences.

The bacterial strain ZN1, from the Tafrant region, was identified as *Enterobacter hormaechei*, and it was excluded from further investigations because of its potential as a human pathogen (Salimiyan Rizi et al. 2019). The strains ZN3, ZN4 and ZN17 were identified as *Pseudomonas* belonging to the species *Pseudomonas frederiksbergensis, Pseudomonas koreensis*, and *Pseudomonas moraviensis*, respectively. The strains ZN12 and ZNSp3 were classified as *Bacillus halotolerans* and *Bacillus pumilus*, while ZN8 and ZN11 were identified as *Streptomyces endophyticus* and *Stenotrophomonas* sp.

Three *Bacillus* species (*Bacillus pumilus*, *Bacillus cereus*, and *Bacillus subtilis*) were selected from the Fez region (ZF1, ZF4B, and ZFSp5) together with four strains identified as *Arthrobacter globiformis, Virgibacillus proomii, Kocuria carniphila*, and *Peribacillus frigoritolerans* (ZF11, ZF13, ZF16, and ZFSp2). Finally, the six bacterial strains isolated from Chichaoua region (ZS7, ZS8, ZSC, ZSSp2, ZSSp3, and ZSSp5) were identified as *Streptomyces venezuelae, Citricoccus* sp., *Terribacillus aidingensis, B. halotolerans, Priestia megaterium*, and *Acinetobacter johnsonii*.

**Figure 4:**
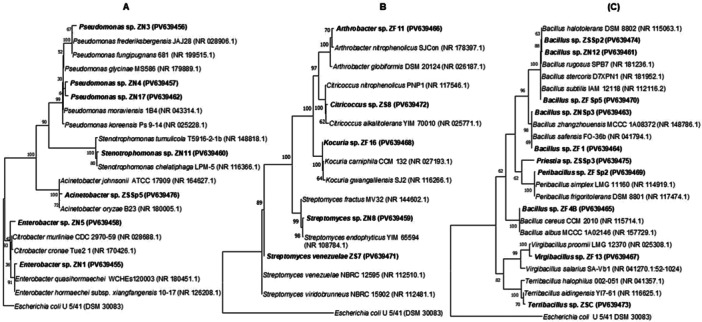
Neighbor‐joining phylogenetic tree based on partial 16S ribosomal RNA (rRNA) gene sequences of seven strains belonging to the subclass Gammaproteobacteria (A), five strains belonging to the subclass Actinobacteria (B), and 10 strains belonging to the subclass Firmicutes (C), isolated from the rhizosphere of *Opuntia ficus‐indica*, along with related species. Isolates are shown in bold. Bootstrap values (from 500 replicates) are indicated as percentages; values below 50% are not displayed. The tree is rooted with *Escherichia coli* U 5/41 (DSM 30083).

Three phylogenetic trees constructed using the Neighbor‐Joining method based on partial 16S rRNA gene sequences of bacterial isolates obtained from the rhizosphere of *O. ficus‐indica* are reported in Figure 4. Bootstrap values greater than 50% are indicated, and the tree was rooted with *E. coli* DSM 30083 as an outgroup. Most isolates showed a high percentage of similarity, ranging between 98.54% and 100.00% with the different grouped strains in the trees: (i) Group (A) comprises seven isolates belonging to Gammaproteobacteria: three have been identified at the genus level as *Pseudomonas*, one as *Stenotrophomonas*, one as *Acinetobacter*, and two as *Enterobacter*. Within this Group *Pseudomonas* isolates (ZN3, ZN4, and ZN17) clustered closely with *P. fluorescens* and *Pseudomonas putida*; (ii) Group (B) includes five isolates belonging to the class Actinobacteria: one was identified as *Arthrobacter*, one as *Citricoccus*, one as *Kocuria*, and two as *Streptomyces*; (iii) Group (C) includes 10 isolates from the phylum Firmicutes: six belonging to the genus *Bacillus*, one to *Peribacillus*, one to *Virgibacillus*, one to *Terribacillus*, and one to *Priestia*. All isolates were submitted to GenBank, and their corresponding codes and accession numbers are indicated in Figure 4.

### 3.5 ACC‐Deaminase Assay

None of the tested bacterial strains showed the capability to produce the enzyme ACC deaminase.

### 3.6 Antibiotic Resistance

Fifteen out of 18 bacterial strains analyzed in this study and belonging to species included in the guidelines and tables provided by EUCAST were tested for antibiotic susceptibility. Since EUCAST standards are primarily developed for clinical isolates, the results obtained in this study should be interpreted with caution and mainly used for comparative purposes among environmental isolates rather than for clinical interpretation. *T. aidingensis* ZSC, *V. proomii* ZF13, *Peribacillus frigotolerans* ZFSp2, and *P. megaterium* (ZSSp3), due to their close phylogenetic relationship, were tested alongside *Bacillus* spp. All the bacterial strains except for *B. cereus* ZF4B, *B. subtilis* ZFSp5, *T. aidingensis* ZSC, and *A. johnsonii* ZSSp5 showed one or more antibiotic resistance (Table 3). Moreover, three bacterial isolates out of the 15 considered can be defined as Multidrug Resistance (MDR) since they are resistant to three or more antimicrobial classes (Magiorakos et al. 2012). Therefore, the strains *Bacillus safensis* ZF1 resistant to meropenem (carbapenem), norfloxacin (quinolone), erythromycin (macrolide), and clindamycin (lincosamide); *V. proomii* ZF13 resistant to erythromycin, clindamycin, Linezolid (oxazolidinone), and vancomycin (glycopeptide); *P. frigotolerans* ZFSp2 resistant to norfloxacin, erythromycin, and clindamycin can be defined as MDR (Table 3).

**Table 3:** Antibiotic resistance of the bacterial strains.

Strain	PRL	FEP	CAZ	DOR	MEM	IMP	ATM	CIP	LEV	NOR	AK	TOB	ERY	DA	LNZ	VA
ZN1	24 (S)	32 (S)	24 (S)	31 (S)	31 (S)	29 (S)	29 (S)	30 (S)	31 (S)	29 (S)	**17 (R)**	**15 (R)**	—	—	—	—
ZN3	26 (I)	29 (I)	24 (I)	39 (I)	36 (I)	34 (I)	**15 (R)**	36 (I)	32 (I)	—		22 (S)	—	—	—	—
ZN4	26 (I)	23 (I)	20 (I)	40 (I)	36 (S)	32 (I)	**13 (R)**	**25 (R)**	25 (I)	—	22 (S)	21 (S)	—	—	—	—
ZN5	—	—	—	—	—	—	—	—	—	—	—	—	—	—	—	—
ZN8	—	—	—	—	—	—	—	—	—	—	—	—	—	—	—	—
ZN11	—	—	—	—	—	—	—	—	—	—	—	—	—	—	—	—
ZN12	—	—	—	—	49 (S)	50 (S)	—	39 (I)	37 (I)	35^a^ (S)	—	—	35 (S)	25 (S)	38 (S)	**6 (R)**
ZN17	26 (I)	25 (I)	23 (I)	24 (I)	22 (I)	31 (I)	**11 (R)**	40 (I)	30 (I)	—	29 (S)	25 (S)	—	—	—	—
ZNSp3	—	—	—	—	35 (S)	46 (S)	—	35 (I)	33 (I)	**18** a **(R)**	—	—	31 (S)	18 (S)	**21 (R)**	19 (S)
ZF1	—	—	—	—	**20 (R)**	45 (S)	—	34 (I)	35 (I)	**6** a **(R)**	—	—	**23 (R)**	**12 (R)**	42 (S)	19 (S)
ZF4B	—	—	—	—	25 (S)	47 (S)	—	25 (I)	28 (I)	26^a^ (S)	—	—	27 (S)	24 (S)	39 (S)	17 (S)
ZF11	—	—	—	—	—	—	—	—	—	—	—	—	—	—	—	—
ZF13	—	—	—	—	32 (S)	35 (S)	—	37 (I)	27 (I)	36^a^ (S)	—	—	**6 (R)**	**6 (R)**	**6 (R)**	**6 (R)**
ZF16	—	—	—	—	—	—	—	—	—	—	—	—	—	—	—	—
ZFSp2	—	—	—	—	50 (S)	50 (S)	—	36 (I)	26 (I)	**19** a **(R)**	—	—	**20 (R)**	**10 (R)**	42 (S)	20 (S)
ZFSp5	—	—	—	—	40 (S)	52 (S)	—	24 (I)	34 (I)	30^a^ (S)	—	—	30 (S)	28 (S)	38 (S)	17 (S)
ZS7	—	—	—	—	—	—	—	—	—	—	—	—	—	—	—	—
ZS8	—	—	—	—	—	—	—	—	—	—	—	—	—	—	—	—
ZSC	—	—	—	—	41 (S)	52 (S)	—	31 (I)	30 (I)	26^a^ (S)	—	—	35 (S)	21 (S)	36 (S)	22 (S)
ZSSp2	—	—	—	—	32 (S)	45 (S)	—	37 (I)	36 (I)	23^a^ (S)	—	—	34 (S)	22 (S)	30 (S)	**8 (R)**
ZSSp3	—	—	—	—	38 (S)	45 (S)	—	26 (I)	28 (I)	22^a^ (S)	—	—	**23 (R)**	**12 (R)**	34 (S)	20 (S)
ZSSp5	—	—	—	—	40 (S)	50 (S)	—	38 (I)	36 (S)	—	25 (S)	21 (S)	—	—	—	—

*Note:* R = resistant (in bold), S = sensitive, and I = intermediate, considering the standards reported by European Committee on Antimicrobial Susceptibility Testing (EUCAST 2025).

Abbreviations: AK, amikacin (30 µg); ATM, aztreonam (30 µg); CAZ, ceftazidime (10 µg); CIP, ciprofloxacin (5 µg); DA, clindamycin (2 µg); DOR, doripenem (10 µg); ERY, erythromycin (15 µg); FEP, cefepime (30 µg); IMP, imipenem (10 µg); LEV, levofloxacin (5 µg); LNZ, linezolid (10 µg); MEM, meropenem (10 µg); NOR, norfloxacin (10 µg); PRL, piperacillin (30 µg); TOB, tobramycin (10 µg); VA, vancomycin (5 µg).

a The norfloxacin disk diffusion test can be used to screen for fluoroquinolone resistance. Isolates categorized as screen negative can be reported “susceptible increased exposure” (I) to ciprofloxacin and levofloxacin. Isolates categorized as screen positive can be reported as resistant to ciprofloxacin and levofloxacin.

### 3.7 Evaluation of Wheat and Sorghum Seed Germination Following Inoculation With High‐Performing PGPR Isolates (In Vitro)

To assess the impact of the bacterial strains on early plant developmental stage, seed germination assay was performed on two plant species *T. durum* (Table 4) and *S. bicolor* (Table 5). Twelve bacterial strains out 18 considered for the test, significantly increased the germination percentage of wheat seeds compared with the control. In detail, *P. frederiksbergensis* ZN3, *Stenotrophomonas* sp., ZN11, *P. moraviensis* ZN17, *A. globiformis* ZF11, *B. subtilis* ZFSp5, *T. aidingensis* ZSC, and *P. megaterium* ZSSp3 significantly increased the germination percentage of wheat seeds compared with the control, reaching 80.00% (Table 4). Besides improving seed germination rate, ZN17, and ZFSp5 increased root length, fresh and dry weights, and the shoot length.

**Table 4:** Effect of inoculation with rhizosphere isolates from *Opuntia ficus‐indica* on germination of wheat seeds (*Triticum durum*). Mean *± *Standard errors. Scores with different letters indicate significant differences among bacterial strains based on the Tukey test (*p* < 0.05).

Treatments	GP%	Number of roots	RL (cm)	FWR (mg)	DWR (mg)	SHL (cm)	FWS (mg)	DWS (mg)	GRI	SVLI	SVWI
Control	64.00 ± 4.00^c^	6.00 ± 0.00^a^	3.33 ± 0.57^e^	16.60 ± 0.57^l^	2.96 ± 0.19^fg^	4.03 ± 0.67^de^	34.17 ± 2.14^h^	4.08 ± 0.26^h^	16.33 ± 0.52^def^	473.10 ± 83.00^fg^	449.60 ± 0.00^i^
ZN3	80.00 ± 4.00^a^	5.67 ± 0.58^ab^	5.33 ± 0.42^abcde^	39.07 ± 0.12^d^	3.92 ± 0.20^bcdef^	6.03 ± 0.06^abc^	57.61 ± 2.88^bc^	6.18 ± 0.31^bc^	17.89 ± 0.71^bcde^	908.90 ± 44.60^abc^	806.00 ± 0.00^c^
ZN4	60.00 ± 0.00^c^	5.00 ± 0.00^ab^	3.77 ± 0.23^de^	34.65 ± 1.49^e^	4.37 ± 0.52^bcd^	6.33 ± 0.47^abc^	56.60 ± 0.61^bc^	5.82 ± 0.42^bc^	14.36 ± 1.13^f^	606.00 ± 42.00^def^	611.15 ± 9.94^fg^
ZN11	80.00 ± 4.00^a^	4.00 ± 1.00^b^	6.47 ± 0.47^ab^	32.93 ± 0.23^efg^	3.55 ± 0.18^defg^	7.27 ± 0.75^a^	56.19 ± 2.81^bc^	5.78 ± 0.29^bc^	18.94 ± 0.46^bc^	1098.00 ± 38.70^a^	744.40 ± 0.00^cd^
ZN12	64.00 ± 4.00^c^	5.00 ± 1.00^ab^	6.10 ± 0.10^abc^	28.84 ± 1.13^hi^	4.03 ± 0.25^bcde^	6.97 ± 1.14^ab^	49.92 ± 3.13^cde^	5.43 ± 0.34^cde^	16.00 ± 0.66^ef^	838.90 ± 116.70^bcd^	604.00 ± 0.00^fg^
ZN17	84.00 ± 0.00^a^	5.33 ± 0.58^ab^	6.20 ± 0.30^abc^	56.77 ± 0.97^b^	4.92 ± 0.18^ab^	6.90 ± 0.17^ab^	68.37 ± 0.37^a^	7.01 ± 0.27^a^	21.81 ± 0.42^a^	1100.40 ± 14.55^a^	1001.70 ± 8.15^a^
ZNSp3	37.33 ± 2.31^e^	6.00 ± 0.00^a^	4.37 ± 1.33^bcde^	20.22 ± 0.25^kl^	3.71 ± 0.32^cdef^	3.57 ± 0.70^e^	35.17 ± 3.68^gh^	4.26 ± 0.36^gh^	8.61 ± 0.64^g^	295.20 ± 68.70^g^	269.68 ± 11.56^j^
ZF1	48.00 ± 4.00^d^	4.67 ± 0.58^ab^	5.80 ± 0.61^abcd^	29.22 ± 0.59^ghi^	3.74 ± 0.31^cdef^	6.67 ± 0.29^ab^	53.92 ± 4.51^bc^	5.51 ± 0.46^bc^	10.83 ± 1.10^g^	600.50 ± 88.10^ef^	442.00 ± 0.00^i^
ZF4B	77.33 ± 2.31^ab^	5.67 ± 0.58^ab^	7.07 ± 0.90^a^	45.31 ± 0.33^c^	5.47 ± 0.16^a^	7.10 ± 0.10^a^	58.60 ± 1.72^b^	6.35 ± 0.19^b^	18.50 ± 0.58^bcd^	1094.80 ± 64.90^a^	913.20 ± 0.00^b^
ZF11	80.00 ± 0.00^a^	6.00 ± 0.00^a^	4.13 ± 1.46^cde^	18.07 ± 0.12^l^	2.61 ± 0.41^g^	5.73 ± 1.16^abcd^	37.10 ± 0.76^fgh^	4.27 ± 0.26^fgh^	20.06 ± 0.48^ab^	789.00 ± 192.00^cde^	550.70 ± 52.50^gh^
ZF13	76.00 ± 4.00^ab^	5.33 ± 1.16^ab^	4.53 ± 0.06^bcde^	33.02 ± 0.54^efg^	4.40 ± 0.23^bcd^	5.833 ± 0.59^abcd^	50.74 ± 2.67^cd^	5.63 ± 0.30^cd^	19.42 ± 1.08^bc^	789.20 ± 86.50^cde^	760.80 ± 0.00^cd^
ZF16	48.00 ± 4.00^d^	5.67 ± 0.58^ab^	3.40 ± 1.40^e^	29.67 ± 0.58^fghi^	4.04 ± 0.34^bcde^	5.17 ± 1.04^bcde^	42.47 ± 3.55^efg^	4.71 ± 0.39^efg^	10.56 ± 0.71^g^	408.00 ± 87.50^fg^	418.00 ± 0.00^i^
ZFSp2	74.67 ± 2.31^ab^	4.33 ± 0.58^ab^	6.10 ± 0.96^abc^	24.73 ± 0.78^j^	3.21 ± 0.23^efg^	6.20 ± 0.46^abc^	51.51 ± 1.39^bc^	5.49 ± 0.17^bc^	17.72 ± 0.49^cde^	917.20 ± 84.80^abc^	648.57 ± 10.80^ef^
ZFSp5	84.00 ± 4.00^a^	5.33 ± 0.58^ab^	4.97 ± 0.60^abcde^	33.45 ± 1.60^ef^	3.63 ± 0.17^def^	5.93 ± 0.32^abcd^	54.37 ± 2.59^bc^	5.41 ± 0.26^bc^	18.86 ± 0.61^bc^	914.10 ± 37.80^abc^	758.00 ± 0.00^cd^
ZS8	68.00 ± 4.00^bc^	5.33 ± 0.58^ab^	5.33 ± 0.49^abcde^	31.26 ± 1.84^efgh^	3.77 ± 0.22^cdef^	7.20 ± 0.46^a^	52.06 ± 3.07^bc^	5.79 ± 0.34^bc^	14.42 ± 0.42^f^	853.30 ± 80.90^bc^	648.40 ± 0.00^ef^
ZSC	80.00 ± 0.00^a^	5.33 ± 0.58^ab^	5.80 ± 0.36^abcd^	29.407 ± 0.46^ghi^	3.77 ± 0.53^cdef^	7.20 ± 0.35^a^	52.97 ± 0.44^bc^	5.61 ± 0.41^bc^	20.06 ± 0.26^ab^	1040.00 ± 8.00^ab^	750.10 ± 74.70^cd^
ZSSp2	76.00 ± 4.00^ab^	4.67 ± 0.58^ab^	5.37 ± 0.40^abcde^	26.43 ± 1.39^ij^	3.40 ± 0.18^defg^	4.63 ± 0.65^cde^	32.73 ± 1.73 ^h^	3.56 ± 0.19^h^	18.86 ± 0.77^bc^	757.20 ± 39.80^cde^	528.00 ± 0.00^h^
ZSSp3	80.00 ± 0.00^a^	6.00 ± 0.00^a^	4.37 ± 0.38^bcde^	23.26 ± 0.99^jk^	3.86 ± 0.70^cdef^	6.47 ± 0.55^abc^	43.57 ± 0.39^def^	5.02 ± 0.21^def^	19.22 ± 0.67^bc^	866.70 ± 18.50^abc^	710.90 ± 40.00^de^
ZSSp5	68.00 ± 4.00^bc^	5.33 ± 0.58^ab^	5.30 ± 0.20^abcde^	65.69 ± 3.87^a^	4.69 ± 0.28^abc^	6.93 ± 0.15^ab^	59.04 ± 3.48^b^	6.05 ± 0.36^b^	15.47 ± 1.17^f^	831.20 ± 33.30^bcde^	728.40 ± 0.00^d^

Abbreviations: DWR, dry weight of roots; DWS, dry weight of shoots; FWR, fresh weight of roots; FWS, fresh weight of shoots; GP%, germination percentage; GRI, germination rate index; RL, root length; SHL, shoot length; SVLI, seedling vigor length index; SVWI, seedling vigor weight index.

**Table 5:** Effect of inoculation with rhizosphere isolates from *Opuntia ficus‐indica* on germination of sorghum seeds (*Sorghum bicolor*). Mean ± Standard errors. Scores with different letters indicate significant differences among bacterial strains based on the Tukey test (*p* < 0.05).

Treatments	GP%	RL (cm)	FWR (mg)	DWR (mg)	SHL (cm)	FWS (mg)	DWS (mg)	GRI	SVLI	SVWI
Control	87.17 ± 1.30^a^	10.26 ± 0.25^ab^	14.60 ± 1.84^a^	2.60 ± 0.10^a^	5.15 ± 0.24^b^	37.30 ± 1.73^ab^	4.05 ± 0.30^bc^	93.09 ± 1.13^a^	304.51 ± 14.61^a^	131.83 ± 11.25^ab^
ZN3	86.92 ± 4.63^a^	8.15 ± 0.84^abc^	12.04 ± 0.27^a^	2.33 ± 0.15^a^	5.58 ± 0.69^ab^	44.08 ± 8.80^ab^	4.98 ± 0.77^ab^	94.17 ± 5.02^a^	264.25 ± 29.54^ab^	138.02 ± 4.47^ab^
ZN4	80.36 ± 6.28^a^	10.89 ± 0.83^a^	18.20 ± 1.64^a^	2.66 ± 0.14^a^	7.88 ± 0.18^a^	67.63 ± 0.51^a^	6.46 ± 0.11^a^	87.06 ± 6.80^a^	321.08 ± 65.09^a^	155.50 ± 29.55^a^
ZN11	80.32 ± 6.65^a^	6.26 ± 0.26^c^	12.40 ± 0.92^a^	2.16 ± 0.09^a^	2.38 ± 0.37^c^	17.52 ± 2.78^b^	2.31 ± 0.29^c^	83.57 ± 4.83^a^	120.60 ± 25.71^b^	74.77 ± 5.38^b^
ZN12	87.61 ± 2.50^a^	9.26 ± 1.33^abc^	16.15 ± 0.89^a^	2.60 ± 0.23^a^	4.01 ± 0.28^bc^	27.63 ± 2.76^b^	3.59 ± 0.15^bc^	93.06 ± 4.40^a^	250.87 ± 35.32^ab^	115.95 ± 8.70^ab^
ZN17	84.41 ± 2.23^a^	10.80 ± 0.34^a^	16.39 ± 2.21^a^	3.09 ± 0.20^a^	4.76 ± 0.17^b^	33.49 ± 1.14^ab^	4.39 ± 0.15^b^	91.44 ± 2.42^a^	286.03 ± 24.80^ab^	137.48 ± 12.99^ab^
ZNSp3	89.22 ± 3.83^a^	9.27 ± 0.17^abc^	14.31 ± 1.17^a^	2.48 ± 0.07^a^	5.26 ± 0.17^b^	41.51 ± 1.60^ab^	4.53 ± 0.17^b^	96.65 ± 4.14^a^	310.89 ± 13.43^a^	155.44 ± 11.30^ab^
ZF1	81.83 ± 3.47^a^	10.78 ± 0.45^a^	14.62 ± 1.34^a^	2.67 ± 0.30^a^	5.50 ± 0.23^b^	38.71 ± 0.86^ab^	4.43 ± 0.07^b^	88.03 ± 3.84^a^	279.59 ± 18.62^ab^	122.74 ± 13.91^ab^
ZF4B	89.98 ± 1.31^a^	10.03 ± 0.53^ab^	15.13 ± 1.46^a^	2.71 ± 0.09^a^	5.09 ± 0.29^b^	39.79 ± 2.52^ab^	4.60 ± 0.13^b^	96.84 ± 1.87^a^	327.31 ± 21.58^ab^	158.05 ± 5.43^a^
ZF11	88.41 ± 5.84^a^	10.44 ± 0.41^ab^	16.14 ± 0.68^a^	2.81 ± 0.32^a^	4.42 ± 0.70^bc^	36.99 ± 11.25^ab^	3.65 ± 0.22^bc^	95.14 ± 6.14^a^	295.31 ± 18.61^a^	130.09 ± 15.82^ab^
ZF13	88.00 ± 6.11^a^	10.30 ± 0.30^ab^	15.14 ± 2.81^a^	2.57 ± 0.13^a^	5.39 ± 0.16^b^	41.04 ± 1.29^ab^	4.19 ± 0.24^b^	95.33 ± 6.62^a^	306.25 ± 42.05^a^	133.33 ± 23.39^ab^
ZF16	86.67 ± 3.53^a^	9.95 ± 0.34^ab^	17.59 ± 1.35^a^	2.75 ± 0.22^a^	5.23 ± 0.20^b^	41.15 ± 2.66^ab^	4.42 ± 0.37^b^	93.89 ± 3.82^a^	285.69 ± 22.02^ab^	134.37 ± 10.20^ab^
ZFSp2	93.67 ± 2.61^a^	9.36 ± 0.26^abc^	16.23 ± 0.24^a^	2.36 ± 0.10^a^	4.56 ± 0.48^bc^	32.38 ± 4.39^ab^	3.65 ± 0.21^bc^	101.48 ± 2.83^a^	326.12 ± 28.07^a^	140.81 ± 9.80^ab^
ZFSp5	82.67 ± 3.53^a^	8.54 ± 0.75^abc^	13.24 ± 2.08^a^	2.71 ± 0.05^a^	3.84 ± 0.52^bc^	37.12 ± 14.19^ab^	3.76 ± 0.30^bc^	86.44 ± 6.01^a^	213.06 ± 29.90^ab^	110.44 ± 6.89^ab^
ZS8	80.00 ± 4.0^a^	7.18 ± 1.40^bc^	15.21 ± 1.23^a^	2.38 ± 0.23^a^	4.93 ± 0.63^b^	45.36 ± 11.52^ab^	4.27 ± 0.46^b^	86.67 ± 4.33^a^	199.81 ± 46.57^ab^	108.14 ± 18.46^ab^
ZSC	86.55 ± 4.49^a^	10.80 ± 0.75^a^	21.09 ± 1.78^a^	2.96 ± 0.29^a^	4.26 ± 0.46^bc^	30.80 ± 4.72^b^	3.68 ± 0.31^bc^	93.76 ± 4.86^a^	311.48 ± 38.31^a^	136.93 ± 16.44^ab^
ZSSp2	86.82 ± 3.56^a^	8.70 ± 0.14^abc^	16.23 ± 0.57^a^	2.99 ± 0.08^a^	4.05 ± 0.81^bc^	36.44 ± 11.02^ab^	4.24 ± 0.57^b^	93.41 ± 3.76^a^	243.02 ± 19.80^ab^	137.40 ± 11.89^ab^
ZSSp3	90.67 ± 2.67^a^	9.31 ± 0.27^abc^	16.78 ± 4.63^a^	2.69 ± 0.03^a^	4.64 ± 0.13^bc^	34.45 ± 0.60^ab^	4.30 ± 0.24^b^	95.56 ± 3.27^a^	286.99 ± 16.51^ab^	143.68 ± 7.98^ab^
ZSSp5	77.00 ± 1.71^a^	10.33 ± 0.18^ab^	17.91 ± 0.62^a^	2.62 ± 0.14^a^	5.02 ± 0.10^b^	42.92 ± 5.08^ab^	4.27 ± 0.05^b^	82.80 ± 2.40^a^	236.23 ± 4.20^ab^	105.99 ± 2.84^ab^

Abbreviations: DWR, dry weight of roots; DWS, dry weight of shoots; FWR, fresh weight of roots; FWS, fresh weight of shoots; GP%, germination percentage; GRI, germination rate index; RL, root length; SHL, shoot length; SVLI, seedling vigor length index; SVWI, seedling vigor weight index.

Furthermore, the relative germination indices and seedling vigor indices, based on length and weight, were markedly improved, reflecting faster germination and more vigorous seedlings. In contrast, seeds inoculated with *B. pumilus* ZNSp3 and *V. proomii* ZF13, showed lower germination rates and limited growth both at root and shoot level compared with uninoculated controls. Inoculation with *P. moraviensis* ZN17 and *B. cereus* ZF4B resulted in seed vigor indices that were approximately twice those of the untreated seeds. On the contrary, *B. pumilus* ZNSp3 and *B. safensis* ZF1 reduced the seed vigor index.

The percentage of germination in sorghum seeds was generally higher than that observed in wheat, with values ranging from 80% to 95%, but none of the tested bacterial strains showed a significant effect on seed germination compared with the controls (Table 5). While root length was increased by *P. koreensis* ZN4, *P. moraviensis* ZN17 and *T. aidingensis* ZSC and reduced by *P. frederiksbergensis* ZN3, *Citricoccus* sp. ZS8 and *Stenotrophomonas* sp. ZN11 (Table 5). In contrast, no significant differences in the fresh and dry root weights were observed between controls and bacterial inoculated seeds (Table 5). A positive effect of *P. koreensis* ZN4 was observed on the aerial part with increased dry shoot weight and shoot length compared with control seedlings (Table 5). On the contrary, *Stenotrophomonas* sp. ZN11 strain showed a negative impact on almost all the seedling parameters (Table 5).

## 4 Discussion

The search for sustainable agricultural solutions has increasingly focused on plant‐associated microbiomes, particularly in arid and semiarid environments. In these ecosystems, plants endure severe abiotic stresses and select for microbial communities that promote nutrient acquisition and stress tolerance. Cacti, such as *O. ficus‐indica*, are widely cultivated in marginal lands and arid zones for their fruit and forage value, and represent a promising source of plant‐beneficial microorganisms well adapted to such stressful conditions (Bulgarelli et al. 2013; Fonseca‐García et al. 2016). While the detailed literature analysis of the interactions between *Opuntia* species and beneficial soil microorganisms is the topic of another work (Angeletti et al, submitted manuscript), a research performed on Web of Science (access date April 2, 2026) by crossing the key words “*Opuntia ficus‐indica* AND rhizosphere” resulted in 18 papers and only 3 of them are focused on the culturable fraction of the bacteria living in the rhizosphere of this plant species (Francisco and Itamar 2012; Lee et al. 2023; Shreshtha et al. 2024).

In this study, a total of 77 bacterial strains were isolated from cactus pear rhizosphere cultivated in Morocco in three geographical regions with different climates: a humid region (Tafrant), a semiarid region (Fez), and an arid one (Chichaoua). The proportion of Gram‐positive versus Gram‐negative bacteria changed according to the sampling site, with a similar distribution among bacterial strains isolated in the Tafrant region, a dominance of Gram positive (70.6%) among the strains isolated in the Fez region and a prevalence of Gram positive (60%) among the isolates from the Chichaoua arid region. While there is no absolute rule driving the predominance of Gram‐positive or Gram‐negative bacteria in the rhizosphere, as the community structure ultimately depends on soil type, plant species, climatic conditions, and local resource availability, the rhizosphere is often enriched in fast‐growing, copiotrophic taxa, such as Proteobacteria and Bacteroidetes, which efficiently exploit carbon‐rich root exudates (Ling et al. 2022). This trend often reverses in plants adapted to arid and semiarid environments, where Gram‐positive, spore‐forming taxa such as Actinobacteria and Firmicutes become more prevalent because of their superior capability to tolerate and survive in low water availability (Akhtar et al. 2021; Bulgarelli et al. 2013; Fonseca‐García et al. 2016). Rather than representing a contradiction, this pattern likely reflects a shift in microbial community composition driven by environmental selection pressures typical of water‐limited environments.

All bacterial isolates have been tested for plant‐beneficial activities, demonstrating multiple PGP traits. In our study, 20.78% of isolates solubilized inorganic phosphate, highlighting that this trait is present in a subset of rhizobacteria. This is comparable to the findings of Beshah et al. (2024), who reported that only 23.17% (19 out of 82) of groundnut rhizobacteria solubilize inorganic phosphate. Previously, Chabot et al. (1993) stated that only 20%–40% of the microbial population in the rhizosphere is able to solubilize phosphate. This activity is particularly significant in arid and semiarid regions where phosphorus is a limiting nutrient in soils and is mediated through organic acid secretion, such as gluconic, malic, acetic, α‐ketoglutaric, lactic, formic, citric, and succinic acids (Ding et al. 2021) contributing to lowering soil pH and acting as effective chelators of Ca^2+^ cations, thereby favoring the release of the soluble form by insoluble phosphate (Goswami et al. 2014).

Regarding the mineralization of organic phosphate, mediated by enzymes cleaving the ester bond between phosphate and the organic compound, thereby releasing inorganic phosphate (Beech et al. 2001), which can then be readily absorbed by plant roots, a regional pattern was evident: while alkaline phosphatase dominated in semiarid and arid regions (Fez and Chichaoua), acid phosphatase prevailed in humid region (Tafrant), reflecting local soil pH conditions (7.64 for Tafrant, 8.05 for Fez, and 7.87 for Chichaoua as reported in Zouitane et al. 2025). In fact, in alkaline soils, alkaline phosphatase activity typically exceeds acid phosphatase activity (Eivazi and Tabatabai 1977). Interestingly, some isolates (i.e., ZN8, ZF4B, and ZS1) exhibited multiple PGP traits, such as the ability to solubilize both inorganic and organic phosphorus, highlighting their multifunctional potential. This trait is particularly relevant under field conditions, where the combined action of different mechanisms can enhance nutrient availability and plant performance.

Similarly to phosphorus, nitrogen is abundant in soil, but it is characterized by a low bioavailability, thus representing a limiting nutrient for plants. In this context, nitrogen‐fixing bacteria are an essential source of nitrogen for plants. According to the qualitative data obtained. The highest proportion of nitrogen‐fixing bacteria (75.00%) was observed in the arid Chichaoua region. The higher proportion of nitrogen‐fixing isolates in the arid Chichaoua region likely reflects the strong selective pressure imposed by nutrient‐poor and water‐limited soils. Biological nitrogen fixation is a highly energy‐demanding process that becomes advantageous when mineral nitrogen is scarce, a condition typical of arid environments (Tang et al. 2023; Barrón‐Sandoval et al. 2023). For instance, the proportion of nitrogen‐fixing bacteria in the rhizosphere of Cactus pear cultivated in the Fez region (35.29%) is consistent with the results presented in Santos et al. (2020), reporting that the 37% of bacteria isolated from the rhizosphere of cactus have shown their ability to fix atmospheric nitrogen.

Also, the ammonia production is considered an important trait of rhizobacteria as it can directly promote plant growth (Joseph et al. 2007) and play a role in signaling during plant‐rhizobacteria interactions (Becker et al. 2002). In this case, the highest proportion of bacteria able to release ammonia starting from an organic source of nitrogen was observed in the rhizosphere of Cactus cultivated in the humid Tafrant region (78.26%). The high ammonium release in the humid soil may be associated with higher microbial activity, favored by greater soil moisture, which enhances nutrient cycling and mineralization processes. In fact, findings by Lu et al. (2025) demonstrated that elevated soil moisture is significantly related to increased nitrogen mineralization (+212%) compared with arid soils.

IAA is a key signaling molecule in the regulation of plant development. By modulating root architecture, IAA promotes lateral root formation and root hair elongation, increasing the root surface area and enhancing water and nutrient uptake under drought (Shreshtha et al. 2024). PGPR are recognized for their ability to produce various phytohormones, including IAA, that is considered as the most significant native auxin (Ashrafuzzaman et al. 2009). Our data showed that 44.16% of the isolates were effective IAA producers with an increasing trend from semiarid Fez (35.29%), to humid Tafrant (43.48%), to arid Chichaoua (60.00%) regions. The enrichment of IAA‐producing bacteria in the arid Chichaoua site aligns with previous reports showing that plants in arid or semiarid environments often select for microbial communities with traits that improve stress resilience, including phytohormone synthesis (Boukelloul et al. 2024; Uzma et al. 2025). In fact, IAA produced by rhizosphere bacteria plays a critical role in plant adaptation to water‐limited conditions. Moreover, bacterial IAA can interact with plant hormonal signaling pathways, mitigating the negative effects of osmotic stress on cell division and elongation, and supporting early seedling establishment in arid environments (Uzma et al. 2025). In this way, IAA‐producing bacteria contribute to plant resilience, improving growth and survival in semiarid and arid soils where water availability is a limiting factor. The amount of IAA produced by the strains ranged from 1.63 µg/mL (isolate ZFSp12) to 25.98 µg/mL (isolate ZS6A). These results are consistent with those of Kavamura et al. (2013), who observed that 30% of the bacterial strains isolated from Brazilian cactus species synthesized IAA at levels above 1.0 µg/mL. Similarly, (Santos et al. 2020), demonstrated that rhizobacteria from three cactus species produced IAA at varying concentrations ranging from 29 to 378 µg/ml.

Exopolysaccharide production is a well‐established bacterial strategy to cope with water‐limited conditions. EPS form a hydrated matrix around bacterial cells, reducing desiccation stress and stabilizing cell membranes under osmotic fluctuations (Liu and Yao 2024). In the rhizosphere, EPS contribute to biofilm formation and soil particle aggregation, which also improve water retention in the surrounding soil, indirectly enhancing plant drought tolerance (Bhagat et al. 2021). Drought‐adapted rhizobacteria, particularly those isolated from arid or semiarid soils, produce higher amounts of EPS compared with isolates from humid environments, suggesting that EPS synthesis is a key adaptive trait selected under osmotic stress (Caruso et al. 2018; Ferioun et al. 2023). Consequently, EPS‐producing bacteria in the rhizosphere serve a dual function: promoting their own survival and contributing to plant resilience under drought conditions. The results obtained in this work highlighted that 45.45% of the isolates are EPS producers. Interestingly, isolates from the humid Tafrant region exhibited the highest proportion of EPS producers (52.17%), followed closely by arid Chichaoua (50.00%) and semiarid Fez (38.24%). While EPS are often associated with drought tolerance, they also play broader roles in bacterial ecology, including biofilm formation, adhesion to root surfaces, and stabilization of the soil matrix (Pal et al. 1999; Caruso et al. 2018). In the humid Tafrant region, higher EPS production may reflect adaptation to the rhizosphere microenvironment, facilitating root colonization, nutrient uptake, and microbial interactions rather than solely osmotic stress protection. This suggests that the functional role of EPS in rhizosphere bacteria is context‐dependent and not exclusively linked to water limitation. Similarly, the highest frequence of bacterial strains able to grow in the presence of PEG6000 was detected in the rhizosphere of cactus pear cultivated in the humid region of Tafrant (65%) and in the semiarid region Fez (64.7%). However, bacteria able to tolerate water potential below –0.49 MPa were more abundant in the semiarid (Fez) and arid (Chichaoua) regions (23.53% and 15.00%, respectively) compared with the humid region (8.70%). The capability of some isolates to grow at water potentials of −0.49 MPa or lower suggests potential relevance under field conditions typical of arid and semiarid soils. This pattern highlights a clear link between environmental origin and functional traits, supporting the role of arid ecosystems as selective reservoirs of drought‐tolerant PGPR. Levels of bacterial drought tolerance vary widely across studies, largely due to differences in methodological criteria, soil and climate conditions, plant species examined, and the heterogeneous physiological strategies adopted by rhizosphere bacteria to cope with water limitation. As an example, Akhtar et al. (2021) reported that the maximum osmotic pressure tolerated by 10% of the bacterial isolates from wheat rhizosphere in Pakistan did not exceed −0.30 MPa, while other works (Ashry et al. 2022; Beshah et al. 2024) reported resistance of isolates up to −0.73, −1.2, and −2.70 MPa, respectively. For sure, in the context of climate change, where drought events are becoming more frequent and severe (Chapman et al. 2021), leveraging drought‐tolerant rhizosphere bacteria could represent a promising strategy to enhance crop resilience and maintain productivity under water‐limited conditions.

However, it should be considered that the drought tolerance assay performed using PEG6000 simulates osmotic stress conditions, but does not fully reproduce the complex conditions of water limitation in soil, where matric potential, soil structure, nutrient availability, and plant–microbe interactions also play important roles. Further studies under soil and greenhouse conditions would be necessary to confirm the drought tolerance and PGP effects of the selected strains under more realistic environmental conditions.

In general, the proportion of bacterial isolates able to synthesize siderophores was quite high ranging from 65.22% in the rhizosphere cultivated in the Tafrant region to 76.47% in Fez region and 80.00% in Chichaoua region. This regional variability may be linked to the physicochemical characteristics of the soils, particularly iron availability. The Fez and Chichaoua regions, which are characterized by lower concentrations of available iron (14.48 and 39.94 µg/g of soil, respectively), showed a higher proportion of siderophore‐producing isolates, suggesting a microbial adaptation to the iron‐limited environment. In contrast, the soil from the Tafrant region, characterized by a higher assimilable iron content (48.03 µg/g of soil), showed a reduced frequency of siderophore‐producing bacteria (Zouitane et al. 2025). This is very interesting under an applicative point of view since siderophore‐producing strains have been used as seed inoculants to improve plant iron nutrition and suppress pathogens restricting the iron availability for those deleterious fungi that are unable to absorb the ferric iron‐siderophore complex (P. Singh et al. 2022).

The capability to suppress the growth of phytopathogenic fungi by PGPR is also based on the release of secondary metabolites, such as hydrogen cyanide, that inhibit enzymes involved in key metabolic processes. HCN is regarded as a biocidal compound with antifungal activity, released together with a mixture of volatile organic compounds (Babalola 2010). Cyanogenic rhizobacteria are often host‐specific. In fact, HCN produced in the environment can help control weeds, while avoiding harmful effects on the growth of desired plants (Kremer and Souissi 2001; Mejri et al. 2010). The amount of bacterial strains selected in this study and showing this physiological trait was quite low (14 out 77). The prevalence of HCN‐producing isolates described in the literature varies widely across studies. For example, Ruchi et al. (2012) isolated 26 strains of *Pseudomonas* sp. from apple and pear rhizosphere, all of which were HCN‐positive. In contrast, other studies have shown a complete absence of HCN production in rhizospheric isolates from Brazilian cactus (Kavamura et al. 2013) and rice (Wickramasinghe et al. 2021).

*F. solani* is known to cause Cactus rot (Ammar et al. 2004); this disease can cause significant economic losses by reducing both fruit yield and marketable quality, thereby impacting farmers' income and the overall profitability of cactus pear cultivation. For this reason, we decided to assess the in vitro antagonistic potential of the bacterial strains against the mycelial growth of *F. solani*, obtaining heterogeneous results. Only four strains (ZN4, ZNSp5, ZS7, and ZSSp5) showed IRs overcoming 60%, two of them able to produce both siderophore and HCN: one negative for both the physiological activities and one positive only for siderophore synthesis. These findings indicate that inhibition of *F. solani* is not exclusively linked to these specific traits, but may result from a combination of strain‐specific metabolic and biochemical features, such as the synthesis of antibiotics (Ortíz‐Castro et al. 2009), or production of lytic enzymes (protease, chitinase, β‐glucanase, and cellulase) (Grobelak et al. 2015; Akocak et al. 2015; Vejan et al. 2016). Therefore, strong antagonistic activity appears to be limited to specific strains rather than being a common trait among all isolates, and these strains should be considered as candidates for further biocontrol studies.

Following PCA, 22 putative high‐performing strains were selected for further analysis: nine coming from the Tafrant region, seven from the Fez, and six from Chichaoua region. These bacterial strains were then identified by 16S rDNA sequencing. Strains belonging to species known as human opportunistic pathogens (*E. hormaechei* and *E. kobei* from the Tafrant region) were excluded from the screening (Salimiyan Rizi et al. 2019). However, several bacterial strains belonging to species or genera known to behave as PGPR or biocontrol agents, able to form beneficial relationships with plants and to colonize efficiently the rhizosphere (Gamalero et al. 2020; Miljaković et al. 2020; Saha et al. 2016; Sun et al. 2017; G. Wang et al. 2022), such as *Pseudomonas* (three species) and *Bacillus* (six species) were efficiently found in this study. Moreover, other strains were identified as *Peribacillus*, *Virgibacillus*, *Terribacillus*, and *Priestia*, all of which have been reported to contribute to plant growth promotion through mechanisms, such as osmotic stress tolerance, production of extracellular enzymes, and enhancement of nutrient availability (Essghaier et al. 2022; Li et al. 2024; Thakur et al. 2024). Overall, according to the phylogenetic analysis, the rhizosphere of *O. ficus‐indica* is a rich reservoir of taxonomically diverse beneficial microorganisms with strong potential for applications in sustainable agriculture. None of the bacterial strains identified showed the capability to produce ACC deaminase. Our result indicates that ACC deaminase is not a dominant trait in the rhizosphere of *O. ficus‐indica* under the studied conditions, highlighting the relevance of alternative mechanisms of stress mitigation. Moreover, data in the literature highlight that different proportions of ACC‐deaminase producers can be found in bulk soil and rhizosphere according to the soil type, climate conditions, or plant and bacterial species (R. P. Singh et al. 2015). By comparing two very recent papers, Ferreira et al. (2025) detected ACC‐deaminase activity in only 32 out of 213 strains isolated from *Brachiaria*, while Alonazi et al. (2025) found positive for this enzyme production 75% of 66 bacterial strains isolated from the rhizosphere of wild *Acacia* sp., mangrove (*Avicennia marina*), and olive trees in Saudi Arabia were able to synthesize this enzyme.

Besides characterizing plant‐beneficial traits, when the exploitation of the bacterial strains is intended for potential agricultural application, it is essential to evaluate their antibiotic sensitivity/resistance, ensuring both their effectiveness for plant growth promotion and their biosafety in the field. In fact, traditionally, studies on antibiotic mechanisms and resistance focused mainly on human pathogens, but, more recently, attention has shifted to the environment, particularly soil, as a major reservoir of resistance genes. Three bacterial isolates, *B. safensis* ZF1, *V. proomii* ZF13, and *P. frigotolerans* ZFSp2 resulted to be Multi Drug Resistant since they were resistant to three or more antimicrobial classes (Magiorakos et al. 2012). Understanding the antibiotic resistance profiles of these environmental strains is of critical importance, as it enables the evaluation of the soil/rhizosphere as a potential reservoir of resistance genes. This knowledge is essential for assessing the risk of antibiotic resistance spreading among the environment, animals, and humans, in line with the One Health approach. Finally, the effect of the bacterial strains on the early stage of plant development was characterized on germination of wheat and sorghum seeds obtaining contrasting results. While 12 out of 18 bacterial strains significantly increased the germination rate of wheat seeds, some of them also increasing the development of the root and shoot of the seedlings, none of the bacterial isolates induced significant effect on sorghum seed germination percentage. In particular, *P. moraviensis* ZN17 and *B. cereus* ZF4B, having strong phosphate solubilization capacities, and *B. subtilis* ZSSp5, synthesizing high levels of IAA, demonstrated a notable performance in improving wheat seed germination and seedling morphological parameters. The isolate *P. moraviensis* ZN17 also showed a positive effect on root length in sorghum seedlings. A key finding of this study is the species‐specific response to bacterial inoculation, with wheat showing significant improvements in germination and early growth, while sorghum responses were more limited. The partial effect of ZN17 on sorghum root length indicates that some mechanisms, such as nutrient mobilization or phytohormone production, may still exert limited benefits across different plant species, but overall, the responses underline the need to carefully match bacterial inoculants with target crops to maximize growth‐promoting outcomes. These results highlight the importance of selecting PGPR strains in a crop‐specific manner, as their effectiveness may vary depending on the plant species.

This study demonstrates that the rhizosphere of *O. ficus‐indica* cultivated in diverse Moroccan environments harbors a taxonomically and functionally diverse bacterial community with multiple PGP traits. The distribution of specific traits, including phosphate solubilization, nitrogen fixation, IAA and EPS production, siderophore synthesis, and antagonism against phytopathogens, reflected both regional environmental conditions and plant‐driven selection pressures. Importantly, while certain strains showed strong potential to enhance wheat germination and seedling development, the effects were species‐specific, emphasizing the necessity to tailor inoculants to target crops. Moreover, the assessment of antibiotic resistance profiles underscores the need for careful biosafety evaluation when considering the application of these strains in agriculture. The presence of antibiotic resistance may reflect intrinsic resistance in some environmental bacteria, but the possible presence of transferable resistance genes should be carefully evaluated. However, not all isolates identified in this study are intended for direct field application; rather, this work represents a preliminary screening aimed at identifying strains with PGP potential. Further characterization of the most promising strains at the genomic level, are required before the introduction of bacterial strains in agricultural applications. In conclusion, these findings highlight *O. ficus‐indica* as a promising and unexplored reservoir of rhizobacteria suitable for sustainable agricultural practices, particularly under arid and semiarid conditions, and provide a valuable framework for selecting and developing effective, safe, and crop‐specific bacterial inoculants.

## Author Contributions

**Ilham Zouitane:** conceptualization, methodology, investigation, data curation, formal analysis, writing – original draft, writing – review and editing. **Daniela Cristina Campana:** conceptualization, methodology, data curation, formal analysis, investigation, writing – original draft, writing – review and editing. **Patrizia Cesaro:** methodology. **Nadia Massa:** methodology, data curation, writing – original draft, writing – review and editing. **Giorgia Novello:** methodology. **Elisa Gamalero:** writing – original draft, writing – review and editing. **Valeria Todeschini:** methodology. **Mohamed Ferioun:** methodology, formal analysis, visualization. **Khalid Derraz:** supervision, writing – review and editing. **Saad Ibnsouda Koraichi:** visualization, resources. **Naïma El Ghachtouli:** conceptualization, funding acquisition, resources, project administration, supervision, writing – review and editing. **Guido Lingua:** conceptualization, funding acquisition, writing – review and editing, project administration, supervision, resources.

## Ethics Statement

The authors have nothing to report.

## Conflicts of Interest

The authors declare no conflicts of interest.

## Supporting information

Supporting File

## Data Availability

The bacterial 16S rDNA reference sequences of these isolates are available in the NCBI World Wide Web database GenBank under SUB 15320870.
