# Supplementary material for: Characterization of Multitrait Plant Growth‐Promoting Rhizobacteria From Opuntia ficus‐indica in Different Moroccan Climates
Source: Microbiologyopen. 2026 Jun 23;15(3):e70334. doi: 10.1002/mbo3.70334 (PMC13291201; doi:10.1002/mbo3.70334)
Supplement: Supplementary file 1 — Supporting File [file MBO3-15-e70334-s001.docx]

**Title:**

**Characterization of Multi-Trait Plant Growth-Promoting Rhizobacteria from *Opuntia ficus-indica* in Different Moroccan Climates**

**Authors:**

Ilham Zouitane^1*^, Daniela CristinaCampana^2^ ^*^, Patrizia Cesaro^2^, Nadia Massa^2^, Giorgia Novello^2^, Elisa Gamalero^2^, Valeria Todeschini^2^, Mohamed Ferioun^1^, Khalid Derraz^3^, Saad Ibnsouda Koraichi^1^, Naïma El Ghachtouli^1‡^, Guido Lingua^2‡^

^*^ *Equal contribution*

^‡^ *Equal contribution*

**Affiliations:**

^1^Laboratory of Microbial Biotechnology and Bioactive Molecules, Sciences and Technology Faculty, Sidi Mohamed Ben Abdellah University, Fez, Morocco.

^2^Department of Science and Technological Innovation, University of Eastern Piedmont, Alessandria, Italy

**Corresponding author:**

Elisa Gamalero

Email: elisa.gamalero@uniupo.it

**Table S1:** List of antibiotics employed in the antibiotic resistance test.

| LABEL | Antibiotic | Disc content (µg) |
| --- | --- | --- |
| PRL | Piperacillin | 30 |
| FEP | Cefepime | 30 |
| CAZ | Ceftazidime | 10 |
| DOR | Doripenem | 10 |
| MEM | Meropenem | 10 |
| IMP | Imipenem | 10 |
| ATM | Aztreonam | 30 |
| CIP | Ciprofloxacin | 5 |
| LEV | Levofloxacin | 5 |
| NOR | Norfloxacin | 10 |
| AK | Amikacin | 30 |
| TOB | Tobramycin | 10 |
| ERY | Erythromycin | 15 |
| DA | Clindamycin | 2 |
| LNZ | Linezolid | 10 |
| VA | Vancomycin | 5 |

**Table S2:** Morphology and Gram reaction of rhizospheric bacteria from O. ficus-indica in the Tafrant, Fez, and Chichaoua regions of Morocco. Scores with different letters indicate significant differences among bacterial strains based on the Tukey test (p < 0.05).

| Isolates | Macroscopic identification | | | | | | | | Microscopic identification |
| --- | --- | --- | --- | --- | --- | --- | --- | --- | --- |
|  | **Color** | **Contour** | **Opacity** | **Shape** | **Size** | **Relief** | **Surface** | **Consistency** |  |
| ZN1 | Pale beige | Regular | Semi opaque | Round | Non punctiform | Raised | Smooth | Viscous | Rod-Shaped Gram- |
| ZN2 | White | Regular | Opaque | Round | Punctiform | Raised | Smooth | Dry | Filamentous |
| ZN3 | Beige | Regular | Opaque | Round | Non punctiform | Raised | Smooth | Viscous | Rod-Shaped Gram- |
| ZN4 | Beige | Regular | Opaque | Round | Non punctiform | Raised | Smooth | Viscous | Rod-Shaped Gram- |
| ZN5 | Yellow | Regular | Opaque | Round | Non punctiform | Raised | Smooth | Viscous | Rod-Shaped Gram- |
| ZN6 | Orange | Irregular | Opaque | Round | Non punctiform | Crater | Rough | Dry | Cocci-shaped Gram+ |
| ZN7 | Beige | Regular | Opaque | Round | Non punctiform | Crater | Rough | Dry | Coccobacilli-Shaped Gram- |
| ZN8 | White | Irregular | Opaque | Round | Non punctiform | Raised | Rough | Dry | Filamentous |
| ZN9 | Yellow | Regular | Opaque | Round | Punctiform | Raised | Smooth | Viscous | Cocci-shaped Gram+ |
| ZN10 | Pale yellow | Regular | Opaque | Round | Punctiform | Raised | Smooth | Viscous | Rod-Shaped Gram- |
| ZN11 | Dark yellow | Regular | Opaque | Round | Non punctiform | Convex | Smooth | Viscous | Rod-Shaped Gram- |
| ZN12 | Off-white | Irregular | Opaque | Round | Non punctiform | Crater | Rough | Dry | Rod-Shaped Gram+ |
| ZN13 | Beige | Regular | Opaque | Round | Punctiform | Raised | Smooth | Creamy | Cocci-shaped Gram+ |
| ZN14 | Off-white | Regular | Opaque | Round | Non punctiform | Raised | Rough | Dry | Filamentous |
| ZN15 | Orange | Regular | Opaque | Round | Non punctiform | Raised | Smooth | Viscous | Coccobacilli-shaped Gram- |
| ZN16 | Yellow | Regular | Opaque | Round | Punctiform | Raised | Smooth | Viscous | Coccobacilli-shaped Gram- |
| ZN17 | Beige | Regular | Opaque | Round | Non punctiform | Raised | Smooth | Viscous | Rod-Shaped Gram- |
| ZNSp1 | Yellow | Regular | Semi opaque | Round | Non punctiform | Flat | Smooth | Viscous | Rod-Shaped Gram+ |
| ZNSp2 | Off-white | Regular | Opaque | Round | Non punctiform | Raised | Smooth | Viscous | Rod-Shaped Gram+ |
| ZNSp3 | White | Irregular | Opaque | Round | Non punctiform | Flat | Smooth | Viscous | Rod-Shaped Gram+ |
| ZNSp4 | White | Irregular | Opaque | Round | Non punctiform | Raised | Rough and wrinkled | Dry | Rod-Shaped Gram- |
| ZNSp5 | Off-white | Irregular | Opaque | Round | Punctiform | Flat | Smooth | Dry | Rod-Shaped Gram- |
| ZNSp6 | Off-white | Regular | Opaque | Round | Non punctiform | Flat | Smooth | Viscous | Cocci-shaped Gram+ |
| ZF1 | Pale beige | Irregular | Opaque | Round | Non punctiform | Flat | Smooth | Viscous | Rod-Shaped Gram+ |
| ZF2 | Beige | Regular | Opaque | Round | Non punctiform | Flat | Smooth | Viscous | Rod-Shaped Gram- |
| ZF3 | Beige | Regular | Opaque | Round | Non punctiform | Raised | Smooth | Viscous | Rod-Shaped Gram- |
| ZF4A | White | Irregular | Opaque | Round | Non punctiform | Plissé | Wrinkled | Dry | Coccobacilli-shaped Gram- |
| ZF4B | White | Irregular | Opaque | Round | Non punctiform | Flat | Wrinkled | Dry | Rod-Shaped Gram+ |
| ZF4C | Off-white | Regular | Opaque | Round | Punctiform | Raised | Smooth | Viscous | Cocci-shaped Gram+ |
| ZF5 | Beige | Regular | Opaque | Round | Non punctiform | Convex | Smooth | Viscous | Rod-Shaped Gram+ |
| ZF6 | Off-white | Regular | Semi opaque | Round | Punctiform | Raised | Smooth | Viscous | Cocci-shaped Gram+ |
| ZF8 | Pale yellow | Regular | Opaque | Round | Punctiform | Raised | Smooth | Viscous | Cocci-shaped Gram+ |
| ZF9 | White | Regular | Opaque | Round | Punctiform | Raised | Smooth | Viscous | Coccobacilli-shaped Gram- |
| ZF10 | Beige | Regular | Opaque | Round | Non punctiform | Convex | Smooth | Viscous | Coccobacilli-shaped Gram- |
| ZF11 | Off-white | Regular | Opaque | Round | Punctiform | Convex | Smooth | Viscous | Cocci-shaped Gram+ |
| ZF12 | Off-white | Regular | Opaque | Round | Non punctiform | Raised | Smooth | Creamy | Cocci-shaped Gram+ |
| ZF13 | Off-white | Regular | Opaque | Round | Non punctiform | Flat | Smooth | Dry | Rod-Shaped Gram+ |
| ZF14 | Beige | Irregular | Opaque | Round | Punctiform | Raised | Rough | Dry | Rod-Shaped Gram+ |
| ZF15 | Off-white | Regular | Opaque | Round | Punctiform | Raised | Smooth | Viscous | Cocci-shaped Gram+ |
| ZF16 | Pale yellow | Regular | Opaque | Round | Punctiform | Raised | Smooth | Viscous | Cocci-shaped Gram+ |
| ZF17 | Pale yellow | Regular | Opaque | Round | Punctiform | Raised | Smooth | Viscous | Cocci-shaped Gram+ |
| ZF18 | Orange | Regular | Opaque | Round | Punctiform | Raised | Smooth | Viscous | Coccobacilli-shaped Gram- |
| ZF20 | Yellow | Regular | Opaque | Round | Punctiform | Raised | Smooth | Viscous | Cocci-shaped Gram+ |
| ZF21 | Off-white | Regular | Opaque | Round | Punctiform | Raised | Smooth | Dry | Cocci-shaped Gram+ |
| ZF22 | White | Regular | Opaque | Round | Punctiform | Raised | Smooth | Viscous | Cocci-shaped Gram+ |
| ZFSp1 | Yellow | Irregular | Semi opaque | Oval | Non punctiform | Flat | Smooth | Viscous | Rod-Shaped Gram- |
| ZFSp2 | Off-white | Regular | Opaque | Round | Non punctiform | Flat | Rough | Dry | Rod-Shaped Gram+ |
| ZFSp3 | Off-white | Regular | Opaque | Round | Non punctiform | Flat | Smooth | Viscous | Rod-Shaped Gram+ |
| ZFSp4 | Beige | Irregular | Opaque | Round | Non punctiform | Flat | Smooth | Viscous | Cocci-shaped Gram+ |
| ZFSp5 | Off-white | Irregular | Opaque | Round | Non punctiform | Raised | Rough | Dry | Rod-Shaped Gram+ |
| ZFSp6 | Beige | Regular | Opaque | Round | Non punctiform | Flat | Smooth | Viscous | Coccobacilli-shaped Gram- |
| ZFSp7 | Off-white | Irregular | Opaque | Round | Non punctiform | Flat | Smooth | Dry | Coccobacilli-shaped Gram- |
| ZFSp8 | Off-white | Regular | Opaque | Round | Punctiform | Convex | Rough | Viscous | Coccobacilli-shaped Gram- |
| ZFSp9 | Pale pink | Irregular | Opaque | Round | Non punctiform | Flat | Smooth | Viscous | Rod-Shaped Gram+ |
| ZFSp10 | Off-white | Regular | Opaque | Round | Punctiform | Raised | Smooth | Viscous | Rod-Shaped Gram+ |
| ZFSp11 | Pale pink | Regular | Opaque | Round | Punctiform | Convex | Smooth | Viscous | Rod-Shaped Gram+ |
| ZFSp12 | Off-white | Regular | Opaque | Round | Punctiform | Raised | Smooth | Viscous | Rod-Shaped Gram+ |
| ZS1 | Pale pink | Regular | Opaque | Round | Punctiform | Raised | Smooth | Viscous | Coccobacilli-shaped Gram- |
| ZS2 | Dark pink | Regular | Opaque | Round | Punctiform | Raised | Smooth | Viscous | Cocci-Shaped Gram- |
| ZS3 | Yellow | Regular | Opaque | Round | Non punctiform | Raised | Smooth | Viscous | Cocci-Shaped Gram+ |
| ZS4 | Orange | Regular | Opaque | Round | Non punctiform | Flat | Smooth | Viscous | Rod-Shaped Gram+ |
| ZS5 | Yellow | Regular | Opaque | Round | Punctiform | Raised | Smooth | Viscous | Coccobacilli-shaped Gram- |
| ZS6A | Off-white | Regular | Opaque | Round | Punctiform | Raised | Smooth | Dry | Cocci-shaped Gram+ |
| ZS6B | Beige | Regular | Opaque | Round | Non punctiform | Raised | Smooth | Creamy | Rod-Shaped Gram- |
| ZS7 | Beige | Irregular | Opaque | Round | Non punctiform | Flat | Rough | Dry | Filamentous |
| ZS8 | Pale yellow | Regular | Opaque | Round | Punctiform | Convex | Smooth | Viscous | Cocci-shaped Gram+ |
| ZS9 | Yellow | Irregular | Opaque | Round | Non punctiform | Flat | Rough | Dry | Coccobacilli-shaped Gram- |
| ZS10 | Off-white | Regular | Semi opaque | Round | Punctiform | Raised | Smooth | Viscous | Cocci-Shaped Gram- |
| ZS11 | White | Regular | Opaque | Round | Punctiform | Raised | Smooth | Dry | Filamentous |
| ZSC | Off-white | Regular | Opaque | Round | Non punctiform | Flat | Smooth | Viscous | Rod-Shaped Gram+ |
| ZSD | White | Regular | Opaque | Round | Non punctiform | Flat | Wrinkled | Dry | Rod-Shaped Gram- |
| ZSSp1 | Yellow | Regular | Semi opaque | Round | Non punctiform | Flat | Smooth | Viscous | Rod-Shaped Gram+ |
| ZSSp2 | White | Irregular | Opaque | Round | Non punctiform | Raised | Wrinkled and rough | Dry | Rod-Shaped Gram+ |
| ZSSp3 | Off-white | Regular | Opaque | Round | Non punctiform | Flat | Smooth | Viscous | Rod-Shaped Gram+ |
| ZSSp4 | Beige | Regular | Opaque | Round | Non punctiform | Raised | Smooth | Viscous | Rod-Shaped Gram+ |
| ZSSp5 | Off-white | Regular | Opaque | Round | Non punctiform | Raised | Smooth | Viscous | Coccobacilli-shaped Gram- |
| ZSSp6 | Off-white | Regular | Opaque | Round | Non punctiform | Raised | Smooth | Viscous | Cocci-shaped Gram+ |

**Table S3**: Plant Growth-Promoting traits of O. ficus-indica rhizobacteria. Mean + Standard errors. Scores with different letters indicate significant differences among bacterial strains based on the Tukey test (p < 0.05).

| Isolates | PSI | Acid Phosphatase (µmol PNP/h/mL) | Alkaline Phosphatase (µmol PNP/h/mL) | IAA (µg/mL) | EPS | N_2_ | NH_3_ | Siderophore (%) | HCN | Antagonism Index (IR %) | Drought Stress (MPa) |
| --- | --- | --- | --- | --- | --- | --- | --- | --- | --- | --- | --- |
| ZN1 | 3.96 ± 0.35^b^ | 0.1538 ± 0.0011 ^a^ | 0.0806 ± 0.0010 ^h^ | 5.69 ± 0.79 ^hi^ | + | + | + | ND | + | 32.54 ± 0.89 ^f^ | -0.05 ± 0.00 ^d^ |
| ZN2 | ND | ND | ND | ND | + | - | - | ND | - | ND | ND |
| ZN3 | 4.38 ± 0.41^a^ | ND | 0.0106 ± 0.0005 ^tuvwxy^ | 10.09 ± 0.59 ^f^ | - | + | + | 23.22 ± 0.59 ^rs^ | + | 31.11 ± 0.47 ^f^ | -0.15 ± 0.00 ^c^ |
| ZN4 | 3.78 ± 0.21^b^ | 0.0334 ± 0.0006 ^e^ | 0.0108 ± 0.0000 ^stuvwx^ | 4.89 ± 0.15 ^hijk^ | - | - | + | 26.18 ± 1.17 ^qr^ | + | 62.69 ± 0.84 ^ab^ | -0.05 ± 0.00 ^d^ |
| ZN5 | 2.79 ± 0.04^d^ | 0.0041 ± 0.0001 ^hi^ | 0.0083 ± 0.0001 ^uvwxyz^ | 23.43 ± 1.11 ^b^ | - | - | + | 13.88 ± 0.28 ^uv^ | - | 53.33 ± 5.77 ^c^ | ND |
| ZN6 | ND | ND | 0.0096 ± 0.0001 ^tuvwxy^ | 3.11 ± 0.60 ^lmn^ | - | - | + | ND | - | 16.03 ± 0.70 ^ij^ | ND |
| ZN7 | ND | ND | ND | 18.87 ± 0.84 ^c^ | - | + | - | 78.19 ± 0.63 ^h^ | - | ND | ND |
| ZN8 | 2.78 ± 0.17^d^ | 0.0735 ± 0.0044 ^c^ | 0.3160 ± 0.0096 ^c^ | 1.28 ± 0.13 ^qrs^ | - | + | + | 52.77 ± 0.51 ^l^ | - | 26.43 ± 0.52 ^g^ | ND |
| ZN9 | ND | ND | 0.0109 ± 0.0003 ^stuvwx^ | ND | + | + | + | 20.65 ± 0.60 ^st^ | - | 26.19 ± 1.30 ^g^ | -0.49 ± 0.00 ^b^ |
| ZN10 | ND | 0.0065 ± 0.0002 ^gh^ | 0.0225 ± 0.0006 ^mnop^ | ND | - | - | + | 27.51 ± 1.34 ^pqr^ | - | ND | -0.05 ± 0.00 ^d^ |
| ZN11 | ND | ND | 0.3390 ± 0.0106 ^b^ | 0.95 ± 0.02 ^qrs^ | + | - | + | 100.00 ± 0.00 ^d^ | - | 50.00 ± 0.29 ^cde^ | ND |
| ZN12 | 1.18 ± 0.07^gh^ | 0.0032 ± 0.0002 ^hi^ | 0.1161 ± 0.0050 ^f^ | 6.03 ± 0.21 ^gh^ | + | + | + | 58.28 ± 0.38 ^k^ | - | 33.97 ± 1.26 ^f^ | -0.15 ± 0.00 ^c^ |
| ZN13 | ND | 0.0001 ± 0.0000 ^i^ | 0.0225 ± 0.0009 ^mnop^ | ND | - | + | + | ND | + | 15.07 ± 0.51 ^jk^ | -0.15 ± 0.00 ^c^ |
| ZN14 | ND | ND | ND | 4.34 ± 0.26 ^ijkl^ | - | + | + | 14.89 ± 5.22 ^uv^ | - | ND | ND |
| ZN15 | ND | 0.0061 ± 0.0001 ^ghi^ | 0.0186 ± 0.0004 ^pqr^ | ND | - | - | - | ND | - | 32.06 ± 0.65 ^f^ | -0.15 ± 0.00 ^c^ |
| ZN16 | ND | ND | 0.0399 ± 0.0002 ^j^ | 5.60 ± 1.42 ^hi^ | + | - | + | 25.32 ± 0.89 ^rs^ | - | ND | -0.05 ± 0.00 ^d^ |
| ZN17 | 3.45 ± 0.17^c^ | 0.0240 ± 0.0005 ^f^ | 0.0092 ± 0.0001 ^tuvwxy^ | 3.36 ± 0.49 ^lmn^ | + | - | + | 8.91 ± 1.01 ^wx^ | + | 16.03 ± 0.57 ^ij^ | -0.73 ± 0.00 ^a^ |
| ZNSp1 | ND | ND | ND | ND | - | - | - | ND | - | ND | ND |
| ZNSp2 | ND | ND | 0.0099 ± 0.0001 ^tuvwxy^ | 3.02 ± 0.37 ^lmno^ | + | + | + | ND | - | 27.38 ± 1.21 ^g^ | -0.05 ± 0.00 ^d^ |
| ZNSp3 | 1.29 ± 0.04^gh^ | ND | 0.0148 ± 0.0009 ^qrstu^ | 0.51 ± 0.04 ^rs^ | + | + | + | 100.00 ± 2.85 ^d^ | + | 50.00 ± 1.17 ^cde^ | -0.15 ± 0.00 ^c^ |
| ZNSp4 | ND | ND | 0.0265 ± 0.0006 ^lmno^ | ND | + | + | + | 11.14 ± 0.31 ^vw^ | - | 8.57 ± 0.02 ^m^ | -0.15 ± 0.00 ^c^ |
| ZNSp5 | ND | ND | 0.0128 ± 0.0005 ^rstuvw^ | ND | + | - | - | ND | - | 63.65 ± 0.55 ^a^ | -0.15 ± 0.00 ^c^ |
| ZNSp6 | ND | ND | 0.0162 ± 0.0004 ^pqrst^ | ND | + | + | + | 15.69 ± 0.86 ^uv^ | - | 21.58 ± 0.68 ^h^ | -0.49 ± 0.00 ^b^ |
| ZF1 | 1.67 ± 0.13^f^ | ND | 0.0149 ± 0.0004 ^qrstu^ | 0.68 ± 0.03 ^rs^ | + | + | + | 17.48 ± 1.48 ^tu^ | - | 46.76 ± 0.99 ^e^ | -0.05 ± 0.00 ^d^ |
| ZF2 | ND | ND | ND | 2.86 ± 0.86 ^mnop^ | - | - | - | 90.00 ± 3.56 ^e^ | - | ND | -0.05 ± 0.00 ^d^ |
| ZF3 | ND | ND | 0.0097 ± 0.0001 ^tuvwxy^ | ND | - | - | - | 91.01 ± 1.22 ^e^ | - | 14.07 ± 0.61 ^jk^ | -0.15 ± 0.00 ^c^ |
| ZF4A | 1.47 ± 0.26^fg^ | ND | 0.0415 ± 0.0006 ^j^ | ND | + | - | - | ND | - | ND | -0.15 ± 0.00 ^c^ |
| ZF4B | 2.50 ± 0.20^de^ | 0.0849 ± 0.0001 ^b^ | 0.2586 ± 0.0059 ^d^ | 0.51 ± 0.03 ^rs^ | + | + | + | 6.11 ± 0.32 ^x^ | + | 33.33 ± 1.22 ^f^ | ND |
| ZF4C | ND | ND | 0.0039 ± 0.0001 ^xyzaaab^ | ND | + | - | + | 7.29 ± 1.26 ^wx^ | - | 34.07 ± 1.28 ^f^ | ND |
| ZF5 | ND | ND | 0.0051 ± 0.0000 ^xyzaaab^ | ND | - | - | + | 27.49 ± 0.45 ^pqr^ | - | ND | -0.15 ± 0.00 ^c^ |
| ZF6 | ND | ND | 0.0061 ± 0.0001 ^wxyzaaab^ | ND | - | - | - | 49.66 ± 0.69 ^lm^ | - | ND | ND |
| ZF8 | ND | ND | 0.0096 ± 0.0000 ^tuvwxy^ | ND | - | - | - | 50.78 ± 0.69 ^lm^ | - | ND | ND |
| ZF9 | ND | ND | ND | ND | - | - | - | 94.11 ± 4.18 ^e^ | - | ND | ND |
| ZF10 | ND | ND | 0.0099 ± 0.0001 ^tuvwxy^ | ND | - | - | + | 90.00 ± 1.95 ^e^ | - | ND | -0.05 ± 0.00 ^d^ |
| ZF11 | ND | ND | 0.0036 ± 0.0000 ^yzaaab^ | 10.50 ± 0.74 ^ef^ | + | + | + | 100.00 ± 1.80 ^d^ | - | 33.08 ± 0.50 ^f^ | -0.49 ± 0.00 ^b^ |
| ZF12 | ND | ND | 0.0075 ± 0.0000 ^vwxyzaa^ | 13.25 ± 0.63 ^d^ | - | + | - | 8.98 ± 0.92 ^wx^ | - | ND | -0.15 ± 0.00 ^c^ |
| ZF13 | ND | 0.0034 ± 0.0001 ^hi^ | 0.0066 ± 0.0000 ^wxyzaaab^ | ND | - | - | - | 249.66 ± 1.11 ^c^ | - | 16.66 ± 1.63 ^ij^ | -0.49 ± 0.00 ^b^ |
| ZF14 | ND | ND | 0.0096 ± 0.0000 ^tuvwxy^ | ND | - | + | + | 80.40 ± 1.58 ^gh^ | - | 50.28 ± 1.11 ^cd^ | -0.05 ± 0.00 ^d^ |
| ZF15 | ND | ND | 0.0216 ± 0.0006 ^nopq^ | 1.21 ± 0.16 ^qrs^ | - | + | - | 71.12 ±1.03 ^i^ | - | 32.00 ± 0.67 ^f^ | ND |
| ZF16 | ND | ND | 0.0324 ± 0.0006 ^kl^ | 5.71 ± 0.11 ^hi^ | - | - | - | 272.22 ± 6.05 ^b^ | - | 12.12 ± 0.73 ^kl^ | -0.49 ± 0.00 ^b^ |
| ZF17 | ND | ND | 0.0108 ± 0.0004 ^stuvwx^ | ND | - | - | - | 65.60 ± 0.92 ^j^ | - | ND | -0.73 ± 0.00 ^a^ |
| ZF18 | ND | ND | 0.0065 ± 0.0000 ^wxyzaaab^ | 5.45 ± 0.67 ^hi^ | + | - | + | ND | - | ND | ND |
| ZF20 | ND | ND | 0.0096 ± 0.0001 ^tuvwxy^ | 5.84 ± 0.11 ^h^ | - | - | - | 39.98 ± 0.02 ^n^ | - | 10.55 ± 0.69 ^lm^ | -0.15 ± 0.00 ^c^ |
| ZF21 | 1.28 ± 0.08^gh^ | ND | ND | ND | - | - | + | 51.34 ± 1.53 ^lm^ | - | ND | ND |
| ZF22 | 2.32 ± 0.07^e^ | 0.0077 ± 0.0000 ^gh^ | 0.0129 ± 0.0002 ^rstuvw^ | ND | - | - | + | 47.00 ± 0.02 ^m^ | - | 15.00 ± 1.17 ^jk^ | ND |
| ZFSp1 | ND | ND | 0.0018 ± 0.0000 ^zaaab^ | ND | - | - | - | 33.45 ± 0.51 ^o^ | - | ND | ND |
| ZFSp2 | ND | ND | 0.0140 ± 0.0002 ^rstuv^ | 11.69 ± 0.74 ^e^ | + | + | + | ND | - | ND | -0.49 ± 0.00 ^b^ |
| ZFSp3 | ND | ND | 0.0072 ± 0.0000 ^vwxyzaaab^ | ND | - | - | - | 51.38 ± 0.55 ^lm^ | - | 15.55 ± 1.02 ^jk^ | ND |
| ZFSp4 | ND | ND | 0.1158 ± 0.0041 ^f^ | ND | + | + | - | ND | - | 19.42 ± 0.83 ^hi^ | -0.15 ± 0.00 ^c^ |
| ZFSp5 | 1.12 ± 0.13^h^ | ND | 0.0687 ± 0.0003 ^i^ | 2.87 ± 0.33 ^mnop^ | + | + | + | 22.95 ± 0.99 ^rs^ | - | 53.24 ± 1.27 ^c^ | -0.73 ± 0.00 ^a^ |
| ZFSp6 | ND | ND | 0.0279 ± 0.0001 ^klmn^ | 1.18 ± 0.34 ^qrs^ | + | - | + | 46.56 ± 1.61 ^m^ | - | 60.83 ± 1.50 ^ab^ | -0.15 ± 0.00 ^c^ |
| ZFSp7 | ND | ND | 0.0637 ± 0.0001 ^i^ | ND | - | - | + | 24.74 ± 0.53 ^rs^ | + | 49.63 ± 1.39 ^de^ | -0.15 ± 0.00 ^c^ |
| ZFSp8 | ND | ND | 0.0179 ± 0.0001 ^pqrs^ | 1.53 ± 0.27 ^pqr^ | + | - | - | ND | + | 27.33 ± 1.20 ^g^ | ND |
| ZFSp9 | ND | ND | 0.0062 ± 0.0001 ^wxyzaaab^ | ND | - | + | - | 32.80 ± 0.71 ^o^ | + | ND | -0.49 ± 0.00 ^b^ |
| ZFSp10 | ND | ND | 0.0279 ± 0.0005 ^klmn^ | ND | - | + | + | ND | - | ND | -0.73 ± 0.00 ^a^ |
| ZFSp11 | ND | ND | 0.0085 ± 0.0000 ^uvwxyz^ | 3.60 ± 0.88 ^klmn^ | + | - | + | ND | - | 26.42 ± 1.21 ^g^ | -0.15 ± 0.00 ^c^ |
| ZFSp12 | ND | ND | 0.0098 ± 0.0000 ^tuvwxy^ | 1.63 ± 0.20 ^opqr^ | + | + | + | ND | - | 33.61 ± 1.27 ^f^ | -0.15 ± 0.00 ^c^ |
| ZS1 | ND | 0.0488 ± 0.0002 ^d^ | 0.6989 ± 0.0032 ^a^ | 7.48 ± 0.74 ^g^ | + | + | - | 51.01 ± 1.03 ^lm^ | - | 14.44 ± 1.24 ^jk^ | ND |
| ZS2 | ND | ND | 0.0059 ± 0.0000 ^wxyzaaab^ | 2.29 ± 0.54 ^nopq^ | - | - | + | 59.51 ± 0.44 ^k^ | - | 32.36 ± 0.92 ^f^ | ND |
| ZS3 | ND | ND | 0.0198 ± 0.0001 ^opqr^ | 3.83 ± 0.74 ^jklm^ | - | + | + | 100.00 ± 0.00 ^d^ | - | ND | -0.15 ± 0.00 ^c^ |
| ZS4 | ND | ND | 0.0089 ± 0.0000 ^tuvwxyz^ | ND | - | - | - | 15.67 ± 0.38 ^uv^ | - | 48.30 ± 1.00 ^de^ | ND |
| ZS5 | ND | ND | 0.0071 ± 0.0000 ^vwxyzaaab^ | ND | + | - | - | 7.19 ± 0.75 ^wx^ | - | ND | ND |
| ZS6A | ND | ND | 0.0097 ± 0.0000 ^tuvwxy^ | 25.98 ± 0.74 ^a^ | + | - | - | 100.00 ± 0.00 ^d^ | - | ND | -0.49 ± 0.00 ^b^ |
| ZS6B | ND | ND | 0.0096 ± 0.0000 ^tuvwxy^ | 2.77 ± 0.36 ^mnop^ | + | + | - | 79.72 ± 0.35 ^h^ | - | ND | -0.15 ± 0.00 ^c^ |
| ZS7 | ND | ND | 0.0107 ± 0.0004 ^stuvwxy^ | 2.29 ± 0.54 ^nopq^ | - | + | - | 315.11 ± 0.19 ^a^ | - | 60.06 ± 0.41 ^b^ | -0.15 ± 0.00 ^c^ |
| ZS8 | ND | ND | 0.0006 ± 0.0000 ^aaab^ | 18.11 ± 0.50 ^c^ | - | + | - | 89.79 ± 2.14 ^ef^ | + | ND | -0.05 ± 0.00 ^d^ |
| ZS9 | ND | ND | 0.0189 ± 0.0002 ^pqr^ | 5.10 ± 0.50 ^hij^ | - | + | + | ND | + | 13.98 ± 0.60 ^jkl^ | -0.49 ± 0.00 ^b^ |
| ZS10 | ND | ND | 0.1063 ± 0.0044 ^g^ | ND | - | - | + | ND | - | 31.94 ± 0.82 ^f^ | ND |
| ZS11 | ND | ND | 0.0046 ± 0.0000 ^xyzaaab^ | ND | - | + | + | 100.00 ± 0.00 ^d^ | - | ND | ND |
| ZSC | ND | 0.0117 ± 0.0150 ^g^ | 0.0350 ± 0.0002 ^jk^ | 9.94 ± 0.50 ^f^ | + | + | + | 47.92 ± 0.88 ^lm^ | - | 14.26 ± 0.35 ^jk^ | -0.15 ± 0.00 ^c^ |
| ZSD | ND | ND | 0.0297 ± 0.0004 ^klm^ | ND | - | + | + | 6.69 ± 0.62 ^wx^ | + | 48.43 ± 0.74 ^de^ | -0.15 ± 0.00 ^c^ |
| ZSSp1 | ND | ND | 0.0042 ± 0.0000 ^xyzaaab^ | ND | + | + | - | ND | - | 16.29 ± 0.84 ^ij^ | ND |
| ZSSp2 | 1.42 ± 0.06^fg^ | ND | 0.1278 ± 0.0031 ^e^ | ND | + | + | + | 31.06 ± 1.11 ^opq^ | - | 48.33 ± 0.67 ^de^ | -0.49 ± 0.00 ^b^ |
| ZSSp3 | 1.24 ± 0.05^gh^ | ND | 0.1188 ± 0.0011 ^f^ | 3.11 ± 0.50 ^lmn^ | + | + | + | ND | - | 59.61 ± 1.18 ^b^ | -0.15 ± 0.00 ^c^ |
| ZSSp4 | ND | ND | 0.0096 ± 0.0000 ^tuvwxy^ | 2.80 ± 0.51 ^mnop^ | + | + | - | 49.60 ± 0.63 ^lm^ | - | 16.66 ± 0.34 ^ij^ | ND |
| ZSSp5 | ND | ND | 0.0227 ± 0.0004 ^mnop^ | 22.64 ± 0.51 ^b^ | - | + | - | 84.94 ± 0.92 ^fg^ | + | 62.69 ± 1.36 ^ab^ | -0.05 ± 0.00 ^d^ |
| ZSSp6 | ND | ND | 0.0042 ± 0.0001 ^xyzaaab^ | 4.86 ± 0.51 ^hijk^ | + | + | + | 31.81 ± 1.68 ^op^ | - | ND | ND |

PSI: Phosphate Solubilization Index, IAA: Indole-3-Acetic Acid, EPS: Exopolysaccharides, N2: Nitrogen, NH3: ammonia, HCN: Hydrogen Cyanide. +: positive test, -: negative test, ND: not detected.
